# *TRIM71* Deficiency Causes Germ Cell Loss During Mouse Embryogenesis and Is Associated With Human Male Infertility

**DOI:** 10.3389/fcell.2021.658966

**Published:** 2021-05-13

**Authors:** Lucia A. Torres-Fernández, Jana Emich, Yasmine Port, Sibylle Mitschka, Marius Wöste, Simon Schneider, Daniela Fietz, Manon S. Oud, Sara Di Persio, Nina Neuhaus, Sabine Kliesch, Michael Hölzel, Hubert Schorle, Corinna Friedrich, Frank Tüttelmann, Waldemar Kolanus

**Affiliations:** ^1^Life and Medical Sciences Institute, University of Bonn, Bonn, Germany; ^2^Institute of Reproductive Genetics, University of Münster, Münster, Germany; ^3^Institute of Medical Informatics, University of Münster, Münster, Germany; ^4^Institute of Pathology, University Hospital Bonn, Bonn, Germany; ^5^Institute for Veterinary Anatomy, Histology and Embryology, Justus Liebig University Gießen, Gießen, Germany; ^6^Hessian Centre of Reproductive Medicine (HZRM), Justus Liebig University Gießen, Gießen, Germany; ^7^Department of Human Genetics, Radboud University Medical Center, Nijmegen, Netherlands; ^8^Centre of Reproductive Medicine and Andrology, Institute of Reproductive and Regenerative Biology, University Hospital Münster, Münster, Germany; ^9^Centre of Reproductive Medicine and Andrology, Department of Clinical and Surgical Andrology, University Hospital Münster, Münster, Germany; ^10^Institute of Experimental Oncology, University Hospital Bonn, Bonn, Germany

**Keywords:** TRIM71/LIN-41, infertility/sterility, azoospermia, Sertoli cell-only (SCO) phenotype, primordial germ cells (PGCs), mammalian gonad development, germ cell tumors (GCT), Haystack

## Abstract

Mutations affecting the germline can result in infertility or the generation of germ cell tumors (GCT), highlighting the need to identify and characterize the genes controlling germ cell development. The RNA-binding protein and E3 ubiquitin ligase TRIM71 is essential for embryogenesis, and its expression has been reported in GCT and adult mouse testes. To investigate the role of TRIM71 in mammalian germ cell embryonic development, we generated a germline-specific conditional *Trim71* knockout mouse (cKO) using the early primordial germ cell (PGC) marker *Nanos3* as a Cre-recombinase driver. cKO mice are infertile, with male mice displaying a Sertoli cell-only (SCO) phenotype which in humans is defined as a specific subtype of non-obstructive azoospermia characterized by the absence of germ cells in the seminiferous tubules. Infertility in male *Trim71* cKO mice originates during embryogenesis, as the SCO phenotype was already apparent in neonatal mice. The *in vitro* differentiation of mouse embryonic stem cells (ESCs) into PGC-like cells (PGCLCs) revealed reduced numbers of PGCLCs in *Trim71*-deficient cells. Furthermore, TCam-2 cells, a human GCT-derived seminoma cell line which was used as an *in vitro* model for PGCs, showed proliferation defects upon *TRIM71* knockdown. Additionally, *in vitro* growth competition assays, as well as proliferation assays with wild type and CRISPR/Cas9-generated *TRIM71* mutant NCCIT cells showed that TRIM71 also promotes proliferation in this malignant GCT-derived non-seminoma cell line. Importantly, the PGC-specific markers *BLIMP1* and *NANOS3* were consistently downregulated in *Trim71* KO PGCLCs, *TRIM71* knockdown TCam-2 cells and *TRIM71* mutant NCCIT cells. These data collectively support a role for TRIM71 in PGC development. Last, via exome sequencing analysis, we identified several *TRIM71* variants in a cohort of infertile men, including a loss-of-function variant in a patient with an SCO phenotype. Altogether, our work reveals for the first time an association of *TRIM71* deficiency with human male infertility, and uncovers further developmental roles for TRIM71 in the germline during mouse embryogenesis.

## Introduction

Despite the recent insights into the regulation of mammalian germ cell development and the advances in assisted reproductive technologies, infertility remains a common problem in modern society affecting ∼15% of couples in industrialized countries (Agarwal et al., 2015). Genetic defects during germ cell development can drive infertility and increase susceptibility to germ cell tumors (GCT) (Raman et al., 2005; Looijenga et al., 2010; Zorrilla and Yatsenko, 2013).

Primordial germ cells (PGCs) are the first established germ cell population during embryonic development. In mice, PGC specification is initiated in the post-implantation epiblast at embryonic day (E) 6.25 in response to BMP4 signaling from the extra-embryonic ectoderm (Lawson et al., 1999). BMP4 activates the expression of *Prdm14* (Yamaji et al., 2008) and *Prdm1/Blimp1* (Ohinata et al., 2005). BLIMP1 then activates the expression of *Tfap2c* (Kurimoto et al., 2008), and together BLIMP1, TFAP2C and PRDM14 establish the transcriptional program required for PGC specification (Wang and Cao, 2016) which is completed at E7.5. At this time point, about 40 cells in the proximal epiblast express early PGC-specific markers such as *Nanos3* (Ginsburg et al., 1990; Tsuda et al., 2003). PGCs then migrate to the genital ridges (developing gonads) while slowly proliferating (Tam and Snow, 1981; Anderson et al., 2000). At E10.5, around 1000 PGCs colonize the genital ridges, upregulate late PGC markers such as *Ddx4/Vasa* (Fujiwara et al., 1994; Tanaka et al., 2000) and *Gcna1* (Enders and May, 1994), and undergo significant proliferation before initiating sex determination (Bowles and Koopman, 2010; Ewen and Koopman, 2010). Importantly, PGCs that fail to reach the genital ridges or to further differentiate into gametes are the origin of GCT, which often affect children and young adults (Raman et al., 2005; Looijenga et al., 2010; Zorrilla and Yatsenko, 2013; Pierce et al., 2018). Therefore, unraveling the processes controlling germline development will not only contribute to our understanding of infertility, but may also offer unique opportunities for the treatment of GCT.

Tripartite Motif Containing 71 (TRIM71) belongs to the TRIM-NHL protein family. TRIM71’s versatile domain structural organization (Ecsedi and Grosshans, 2013) enables its role as an E3 ubiquitin ligase (Chen et al., 2012; Nguyen et al., 2017; Ren et al., 2018; Hu et al., 2019) and an mRNA-binding protein (Chang et al., 2012; Loedige et al., 2013; Worringer et al., 2014; Mitschka et al., 2015; Torres-Fernández et al., 2019). TRIM71 function is essential for embryogenesis and its expression is mostly restricted to undifferentiated cells during early proliferative developmental stages, being downregulated in the course of differentiation (Schulman et al., 2005, 2008; Rybak et al., 2009; Cuevas et al., 2015). However, a postnatal *Trim71* expression has also been observed in adult mouse testes (Rybak et al., 2009; Du et al., 2020) as well as in several GCT-derived cell lines (Rybak et al., 2009; Chang et al., 2012; Torres-Fernández et al., 2019), and a recent study has reported a postnatal function for TRIM71 in adult mouse spermatogenesis (Du et al., 2020). Furthermore, RNA sequencing of *Trim71*-deficient mouse embryonic stem cells (ESCs) revealed a decreased expression of genes associated with reproductive processes (Mitschka et al., 2015), suggesting an early developmental role for TRIM71 in the germline.

To elucidate the role of TRIM71 in mammalian embryonic germ cell development, we generated a mouse model with an early germline-specific depletion of *Trim71* driven by the *Nanos3* promoter (*Nanos3*-Cre). Additionally, we employed an *in vitro* approach for the differentiation of wild type and *Trim71*-deficient murine ESCs into PGC-like cells (PGCLCs) in order to study the role of TRIM71 during PGC specification. Furthermore, we used the human GCT-derived seminoma cell line TCam-2 as a surrogate *in vitro* model of PGCs and evaluated cell proliferation upon *TRIM71* knockdown. Additionally, we used the human GCT-derived non-seminoma cell line NCCIT to generate *TRIM71* mutations via CRISPR/Cas9 in order to investigate the role of TRIM71 in the proliferation of pluripotent embryonal carcinoma cells. Last, we used exome sequencing data from infertile men and developed a novel software tool named Haystack to search for novel genetic causes of infertility. Our work here identifies *TRIM71* as a novel gene associated with human male infertility and uncovers a novel role for TRIM71 in the embryonic development of the germline.

## Results

### *Trim71* Is Expressed in Spermatogonial Stem Cells and Is Essential for Mouse Fertility

**FIGURE 1:**
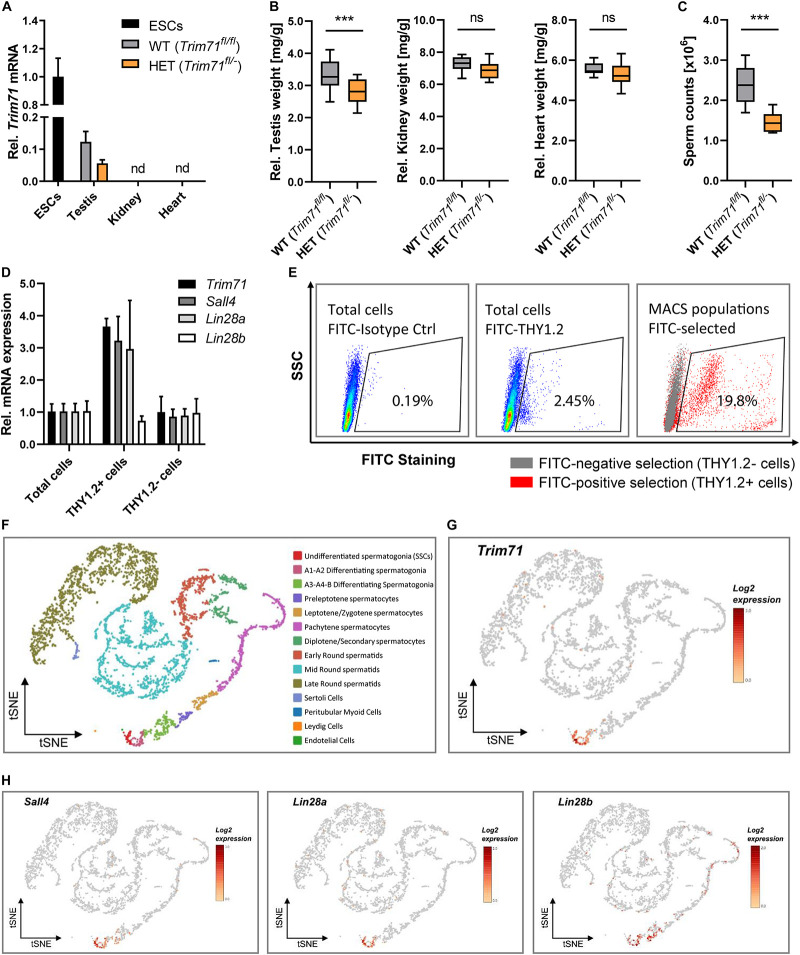
*Trim71* is expressed in spermatogonial stem cells (SSCs) of adult fertile mice. **(A)** qRT-PCR of *Trim71* relative to *Hprt* housekeeping gene in testis, kidney and heart of wild type (WT, *Trim71*^*fl/fl*^) and *Trim71* heterozygous (HET, *Trim71^*fl/*−^*) adult male mice (10–14 weeks old), normalized to *Trim71* relative levels in wild type murine ESCs (nd, not detected). Error bars represent SD (*n* = 3). **(B)** Organ weight [mg] relative to total body weight [g] of testis, kidney and heart of wild type (WT, *Trim71*^*fl/fl*^) and *Trim71* heterozygous (HET, *Trim71*^*fl/*−^) male adult mice (10–14 weeks old). Graphs represent Tukey plots (*n* = 14–16). ***P-value < 0.005, ns, non-significant (unpaired Student’s *t*-test). **(C)** Epididymal sperm counts in wild type (WT, *Trim71*^*fl/fl*^) and *Trim71* heterozygous (HET, *Trim71^*fl/*−^*) male adult mice (10–14 weeks old). ***P-value < 0.005 (unpaired Student’s *t*-test). **(D)** qRT-PCR of *Trim71*, the SSC markers *Sall4* and *Lin28a*, and the differentiating spermatogonia marker *Lin28b*, relative to *Hprt* housekeeping gene in testes cell suspension before (total cells) and after THY1.2 MACS (THY1.2+/− cells). Error bars represent SD (*n* = 3). **(E)** Representative flow cytometry scatter plots of testes cell suspension before (total cells) and after THY1.2 MACS (THY1.2+/− cells). **(F)** tSNE plot (*t*-distributed stochastic neighbor embedding) of single-cell transcriptome data (scRNA-seq) from mouse testes as published by Hermann et al. (2018). Each dot represents a single cell and is colored according to its cluster identity as indicated on the figure key. **(G)** Expression pattern of *Trim71* and **(H)** *Sall4*, *Lin28a* and *Lin28b* projected on the tSNE plot of the mouse scRNA-seq dataset. Red indicates high expression and gray indicates low or no expression, as shown by the figure key. See also Supplementary Figures 1, 2.

Mice carrying a *Trim71* homozygous deletion (KO, *Trim71*^–/–^) die during embryonic development (Supplementary Figure 1A; Schulman et al., 2008; Cuevas et al., 2015). In contrast, *Trim71* heterozygous mice (HET, *Trim71*^*fl/*–^) are viable and fertile, although significantly smaller in length and weight than wild type (WT, *Trim71*^*fl/fl*^) littermates (Supplementary Figures 1B,C). We detected *Trim71* expression in the testes – but not in other organs such as heart or kidney – of wild type and heterozygous adult mice, with *Trim71* mRNA levels decreased in the testes of heterozygous mice (Figure 1A). We then measured the weight of several organs relative to the total body weight, and observed that testes – but neither heart nor kidney – were significantly smaller in heterozygous males compared to wild type males (Figure 1B). Accordingly, sperm counts were significantly reduced in adult heterozygous males (Figure 1C). These data suggested that *Trim71* expression levels are important for male gonad development and revealed a haploinsufficiency of *Trim71* in mice.

**FIGURE 2:**
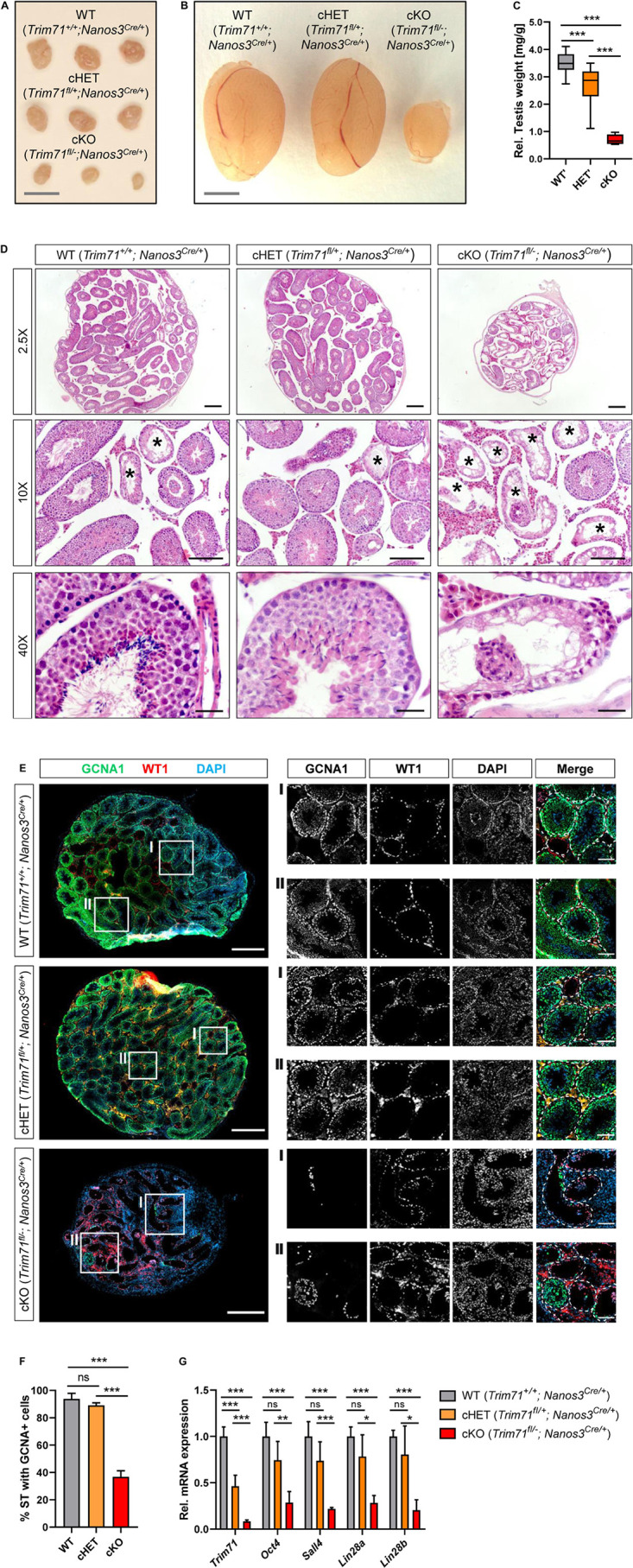
Germline-specific *Trim71* cKO male mice present a Sertoli cell-only (SCO) phenotype. **(A)** Representative images of ovaries and **(B)** testes of adult wild type (WT, *Trim71*^+/+^; *Nanos3*^*Cre/*+^), germline-specific *Trim71* heterozygous (cHET, *Trim71*^*fl/*+^; *Nanos3*^*Cre/*+^) and germline-specific *Trim71* knockout (cKO, *Trim71*^*fl/*−^; *Nanos3*^*Cre/*+^) mice. Scale bars represent 2 mm. **(C)** Testis weight [mg] relative to total body weight [g] of wild type (WT’), *Trim71* heterozygous (HET’) and germline-specific *Trim71* knockout (cKO) mice, summarized from Supplementary Figure 2C, joining several *Nanos3* genotypes per *Trim71* genotype. Error bars represent SD (*n* = 8–29). ***P-value < 0.005 (one-way ANOVA, Tukey’s test). **(D)** Representative H&E stainings on paraffin testes cross-sections from adult wild type (WT, *Trim71*^+/+^; *Nanos3*^*Cre/*+^), germline-specific *Trim71* heterozygous (cHET, *Trim71*^*fl/*+^; *Nanos3*^*Cre/*+^) and germline-specific *Trim71* knockout (cKO, *Trim71*^*fl/*−^; *Nanos3*^*Cre/*+^) mice. Seminiferous tubules with a defective morphology are marked with an asterisk (*) in 10x magnification images. Scale bars represent 200, 100, and 20 μm in 2.5×, 10×, and 40× magnifications, respectively. **(E)** Representative immunofluorescence stainings on testes cryosections from adult wild type (WT, *Trim71*^+/+^; *Nanos3*^*Cre/*+^), germline-specific *Trim71* heterozygous (cHET, *Trim71*^*fl/*+^; *Nanos3*^*Cre/*+^) and germline-specific *Trim71* knockout (cKO, *Trim71*^*fl/*−^; *Nanos3*^*Cre/*+^) mice. Images show co-staining with GCNA1 (germ cells), WT1 (Sertoli cells) and DAPI (nuclei). For each genotype two regions – indicated as I and II – are depicted in higher magnification. Scale bars represent 500 μm for complete testes cross-sections and 100 μm for the magnification images. **(F)** Quantification of seminiferous tubules (ST) containing GCNA+ cells per testis cross-section from **E**, depicted as percentages. Error bars represent SD (*n* = 3). ***P-value < 0.005, ns, non-significant (unpaired Student’s *t*-test). **(G)** qRT-PCR of *Trim71*, the murine SSC markers *Sall4*, *Lin28a* and *Oct4*, and the differentiating spermatogonia marker *Lin28b*, relative to *Hprt* housekeeping gene in whole testis RNA of wild type (WT, *Trim71*^+/+^; *Nanos3*^*Cre/*+^), germline-specific *Trim71* heterozygous (cHET, *Trim71*^*fl/*+^; *Nanos3*^*Cre/*+^) and germline-specific *Trim71* knockout (cKO, *Trim71*^*fl/*−^; *Nanos3*^*Cre/*+^) male adult mice. Error bars represent SD (*n* = 3−6). ***P-value < 0.005; ***P-value < 0.01; *P-value < 0.05; ns, non-significant (unpaired Student’s *t*-test). See also Supplementary Figures 3, 4.

Since TRIM71 is usually expressed in undifferentiated cells, we hypothesized that its expression in mice testes is present in undifferentiated spermatogonia, also known as spermatogonial stem cells (SSCs). To validate this hypothesis, we used wild type testes cell suspensions to enrich SSCs (THY1.2 +) via magnetic-activated cell sorting (MACS) (Liao et al., 2016). We found an increase of *Trim71* mRNA as well as the mRNAs of the known murine SSC-specific markers *Sall4* (Gassei and Orwig, 2013) and *Lin28a* (Zheng et al., 2009) – but not the differentiating spermatogonia marker *Lin28b* (Gaytan et al., 2013) – in the THY1.2 + enriched cell population (Figures 1D,E). This result indicated that *Trim71* expression in adult mouse testes is enriched in SSCs. Indeed, single-cell RNA sequencing analysis of mouse and human adult testicular tissue confirmed a restricted expression of *Trim71* in SSCs (Figures 1F–H and Supplementary Figure 2; Hermann et al., 2018).

To study the function of TRIM71 in male gonad development, a germline-specific Trim71 conditional knockout mouse (cKO) was generated with the Cre recombinase expressed under the promoter of the early PGC marker *Nanos3* (Suzuki et al., 2008; Supplementary Figure 3A). For a first functional evaluation, adult cKO mice (*Trim71*^*fl/*–^*; Nanos3*^*Cre/*+^) were crossed with wild type (*Trim71*^+/+^) mice. *Trim71* deficiency in the germline results in infertility in both sexes, as neither cKO males nor females were able to produce offspring after numerous mating attempts over the course of several months (Supplementary Figure 3B).

### Germline-Specific *Trim71* cKO Male Mice Display a Sertoli Cell-Only (SCO)-Like Phenotype

Macroscopic analysis of cKO male and female reproductive organs revealed a dramatic reduction in testis and ovary size, respectively (Figures 2A–C and Supplementary Figure 3C). Hematoxylin and eosin (H&E) staining of adult male testis cross-sections showed that most seminiferous tubules in cKO testes were lacking signs of spermatogenesis (Figure 2D). Furthermore, immunofluorescence staining of germ cells (GCNA1 Koshimizu et al., 1995; Tanaka et al., 1998) and Sertoli cells (WT1 Mundlos et al., 1993) showed a dramatic reduction of GCNA + cells in cKO mice (Figures 2E,F). Accordingly, the expression of murine SSC-specific markers *Oct4, Sall4* and *Lin28a*, as well as the differentiating spermatogonia marker *Lin28b*, was dramatically reduced in whole testis RNA of cKO mice (Figure 2G), representing the deficit of germ cells in these mice.

This phenotype is reminiscent of a human condition known as Sertoli cell-only (SCO) phenotype, which is a specific type of non-obstructive azoospermia characterized by a total or substantial absence of germ cells in the seminiferous tubules, and whose causes remain mostly unknown (Tüttelmann et al., 2011, 2018; Koc et al., 2019). Our results here suggest that *Trim71* cKO male mice display an SCO-like phenotype with most seminiferous tubules lacking germ cells.

### Infertility in Germline-Specific *Trim71* cKO Male Mice Has an Embryonic Origin

**FIGURE 3:**
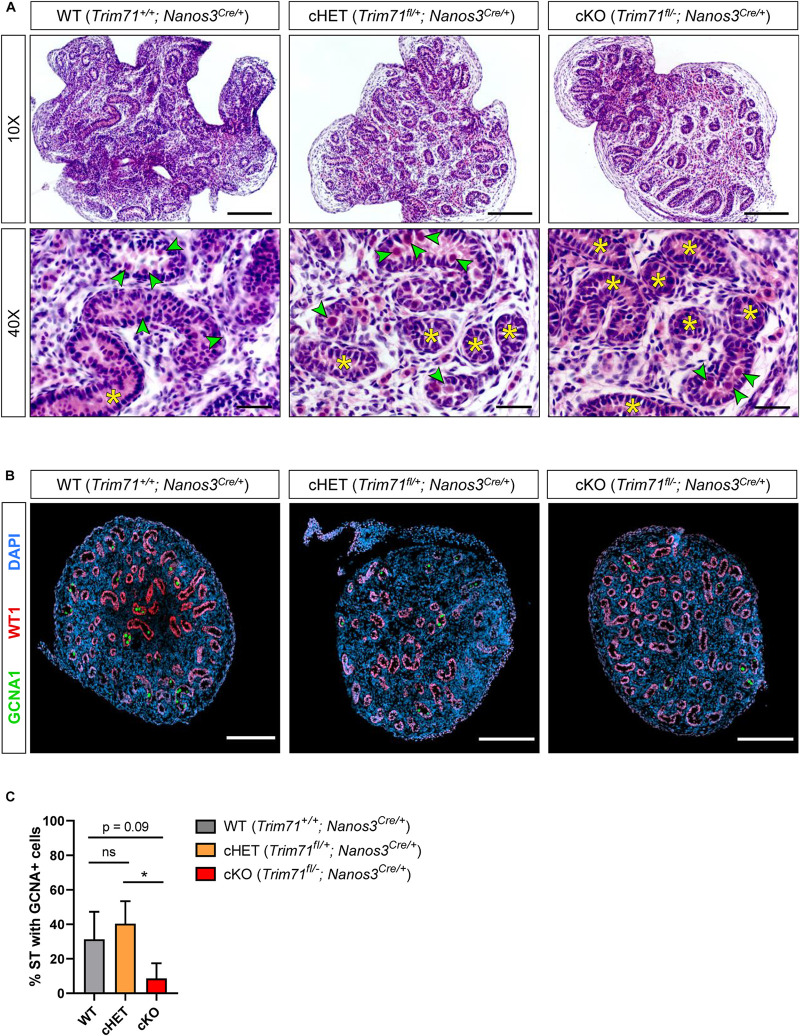
Infertility in germline-specific *Trim71* cKO male mice has an embryonic origin. **(A)** Representative H&E stainings on paraffin testes cross-sections from neonatal (P0.5) wild type (WT, *Trim71*^+/+^; *Nanos3*^*Cre/*+^), germline-specific *Trim71* heterozygous (cHET, *Trim71*^*fl/*+^; *Nanos3*^*Cre/*+^) and germline-specific *Trim71* knockout (cKO, *Trim71*^*fl/*−^; *Nanos3*^*Cre/*+^) male mice. Gonocytes within the seminiferous tubules are marked with a green arrow head, and gonocyte-lacking seminiferous tubules are marked with a yellow asterisk (*) in 40x magnification images. Scale bars represent 100 and 20 μm in 10× and 40× magnifications, respectively. **(B)** Representative immunofluorescence stainings on testes cryo-sections from neonatal (P0.5) wild type (WT, *Trim71*^+/+^; *Nanos3*^*Cre/*+^), germline-specific *Trim71* heterozygous (cHET, *Trim71*^*fl/*+^; *Nanos3*^*Cre/*+^) and germline-specific *Trim71* knockout (cKO, *Trim71*^*fl/*−^; *Nanos3*^*Cre/*+^) male mice. Images show co-staining with GCNA1 (gonocytes), WT1 (Sertoli cells) and DAPI (nuclei). Scale bars represent 200 μm. **(C)** Quantification of seminiferous tubules (ST) containing GCNA+ cells per testis cross-section from **B**, depicted as percentages. Error bars represent SD (*n* = 3). *P-value < 0.05, ns, non-significant (unpaired Student’s *t*-test). See also Supplementary Figures 4–6.

To investigate the origin of infertility in *Trim71* cKO mice, we first analyzed available datasets for the expression of *Trim71* in murine male and female gonads. While *Trim71* expression in males is present in both fetal and adult testes, *Trim71* expression in females is detected in fetal ovaries but not in adult ovaries (Supplementary Figure 4A; Han et al., 2018). These results were confirmed by western blot comparing TRIM71 expression in adult testes and ovaries of wild type and *Trim71* cKO mice (Supplementary Figure 4B). Such an expression pattern may indicate that TRIM71 fulfills embryonic and postnatal functions in the male germline, but only embryonic functions in the female germline. Because a postnatal function of TRIM71 in adult mouse spermatogenesis has already been described (Du et al., 2020), we decided to focus on the possible TRIM71 function during embryonic germline development. As *Nanos3* expression in mice embryos is detected as early as E7.5 during PGC specification (Tsuda et al., 2003), the infertility observed in both male and female *Trim71* cKO mice may reflect a function of TRIM71 in an early stage of germ cell development. Thus, we next analyzed available datasets for *Trim71* expression in murine fetal gonads. The expression of *Trim71* followed a similar pattern for male and female gonads during different mouse developmental stages (Supplementary Figure 4C; Soh et al., 2015; Zhao et al., 2018) which correlated with *Trim71* expression in isolated male and female germ cells at the same stages (Supplementary Figure 4D; Seisenberger et al., 2012). This expression pattern supports a possible role for TRIM71 in germ cell development. In order to determine whether the deficit of germ cells observed in adult *Trim71* cKO male mice derives from defects during embryonic development, we conducted H&E staining and GCNA1-WT1 immunostaining on testis cross-sections from neonatal (P0.5) male mice (Figures 3A,B). Indeed, the SCO-like phenotype previously observed in adult male cKO mice was already apparent in neonatal cKO mice as shown by a reduction in gonocyte-containing seminiferous tubules (Figures 3B,C). These results confirm that *Trim71* expression during embryonic development is required for the generation and/or maintenance of the male germline before birth.

### *In vitro* PGC Specification Reveals Reduced Numbers of PGCLCs Derived From *Trim71*-Deficient ESCs

In order to determine whether *Trim71* deficiency causes PGC specification defects, wild type (WT, *Trim71*^*fl/fl*^) and *Trim71* knockout (KO, *Trim71*^–/–^) murine ESCs (Mitschka et al., 2015) were differentiated into PGC-like cells (PGCLCs) *in vitro* as previously described (Hayashi et al., 2011). In short, ESCs (d0) growing under naïve conditions were primed to epiblast-like cells (EpiLCs) (d2) for two days in monolayer culture and differentiated into PGCLCs (d8) for six more days in the presence of BMP4 while growing as spheroids (Supplementary Figure 5A). ESCs (d0) represent *in vivo* embryonic stem cells of the blastocyst’s inner cell mass at E3.5-4.5 while EpiLCs (d2) represent *in vivo* epiblast stem cells (EpiSCs) at E5.5-6.5, and PGCLCs (d8) correspond to *in vivo* PGCs at E9.5 (Hayashi et al., 2011). Thus, several markers were measured in bulk cell populations at d0, d2 and d8 to monitor the differentiation process (Supplementary Figure 6). Of note, these experiments are rather sensitive to small variations in experimental conditions and need to be interpreted with caution.

Both WT and *Trim71* KO ESCs were primed to EpiLCs as shown by a decrease of the naïve pluripotency marker *Klf4* (Guo et al., 2009; Hayashi et al., 2011) and a simultaneous increase of the primed pluripotency epiblast-specific marker *Dnmt3b* (Hayashi et al., 2011; Veillard et al., 2014) at d2 (Supplementary Figures 6A,B). The subsequent *Dnmt3b* downregulation observed at d8 was indicative of a successful specification induction by BMP4, as the early PGC marker *Prdm14* represses *Dnmt3b* to enable PGC epigenetic reprogramming (Grabole et al., 2013; Seki, 2018). Accordingly, both WT and *Trim71* KO cells showed a significant upregulation of PGC-specific markers at d8 compared to d2 including *Prdm14*, *Prdm1/Blimp1* and their downstream targets *Tfap2c* and *Nanos3* (Supplementary Figures 6C–F). However, the levels of *Blimp1* at d8 were significantly lower in *Trim71* KO cells than in WT cells, an effect that was also apparent – although not significant – for *Nanos3*. Importantly, while an upregulation of the early PGC marker *Nanos3* (Tsuda et al., 2003) was already observable at d8, the expression of the late PGC marker *Ddx4/Vasa* (Fujiwara et al., 1994; Tanaka et al., 2000) was not increased (Supplementary Figure 6G), indicating that the *in vitro*-generated PGCLCs represent *in vivo* PGCs prior to the time point of *Ddx4* expression (E10.5), as expected (Hayashi et al., 2011).

In order to estimate the number of PGCLCs specified from WT and *Trim71* KO ESCs, d8-bulk populations were stained for the surface markers ITGB3/CD61 (PE-Cy7) and SSEA-1/CD15 (APC) – previously reported to unequivocally identify PGCLCs (Hayashi et al., 2011) – and were analyzed by flow cytometry. We found that the number of PGCLCs derived from *Trim71*-deficient ESCs was reduced by about 25% (Supplementary Figures 5B,C). A significant decrease of the PGC-specific marker SSEA-1/CD15 (APC) – but not of ITGB3/CD61 (PE-Cy7) – was also observed in PGCLCs derived from *Trim71*-deficient ESCs (Supplementary Figures 5D,E). However, these stainings do not show the expected distinct PGCLC population (Hayashi et al., 2011), which might be indicative of an incomplete induction.

Of note, *Oct4* is required for the maintenance of PGCs after their specification (Kehler et al., 2004). The highest expression of *Oct4* in themouse embryo is observed in the early blastocyst and decreases progressively in the epiblast upon gastrulation until being restricted to PGCs at E7.5 (Rosner et al., 1990; Ecsedi and Grosshans, 2013). Accordingly, we detected a high *Oct4* expression in ESCs (d0) which significantly decreased upon priming to EpiLCs (d2) and was maintained in PGCLCs (d8) upon specification (Supplementary Figure 6H). A similar pattern was observed for *Trim71* expression in WT cells (Supplementary Figure 6I) with a decrease in its mRNA level after priming (d2) and a sustained expression in PGCLCs (d8). While our results have revealed a moderate reduction in the number of *Trim71*-deficient PGCLCs upon induction, we believe that this cannot fully explain the dramatic phenotype observed in *Trim71* cKO mice. This, together with the sustained *Trim71* expression in PGCLCs at d8, suggests a further role for TRIM71 downstream of PGC specification.

### TRIM71 Deficiency Does Not Impair *in vivo* Migration of PGCs Toward the Genital Ridges

After specification, PGCs migrate along the hindgut toward the genital ridges (Tam and Snow, 1981; Anderson et al., 2000). Thus, we next investigated whether TRIM71 is required for PGC migration *in vivo*. To this end, we used full *Trim71* KO (*Trim71*^–/–^) embryos resulting from the mating of full heterozygous (*Trim71*^*fl*/–^) animals in order to exclude an incomplete *Trim71* deletion at this stage resulting from an insufficient *Nanos3*-driven Cre expression. Wild type (*Trim71*^*fl/fl*^) and full KO (*Trim71*^–/–^) embryos at stages E8.5-8.75 were dissected to isolate the hindgut and alkaline phosphatase (AP)-positive PGCs were stained. PGCs were detected in close proximity to the allantois in both wild type and *Trim71*-deficient embryos (Supplementary Figure 7). Although our AP-stainings do not provide quantitative information, they clearly show that *Trim71* expression is not essential for migration of PGCs toward the genital ridges and furthermore reinforce the notion that no major PGC specification defects are apparent, as already suggested by our PGCLC induction experiments. Thus, TRIM71 likely affects germ cell development at later embryonic stages.

### TRIM71 Controls Proliferation in the PGC-Like *in vitro* Model Cell Line TCam-2

**FIGURE 4:**
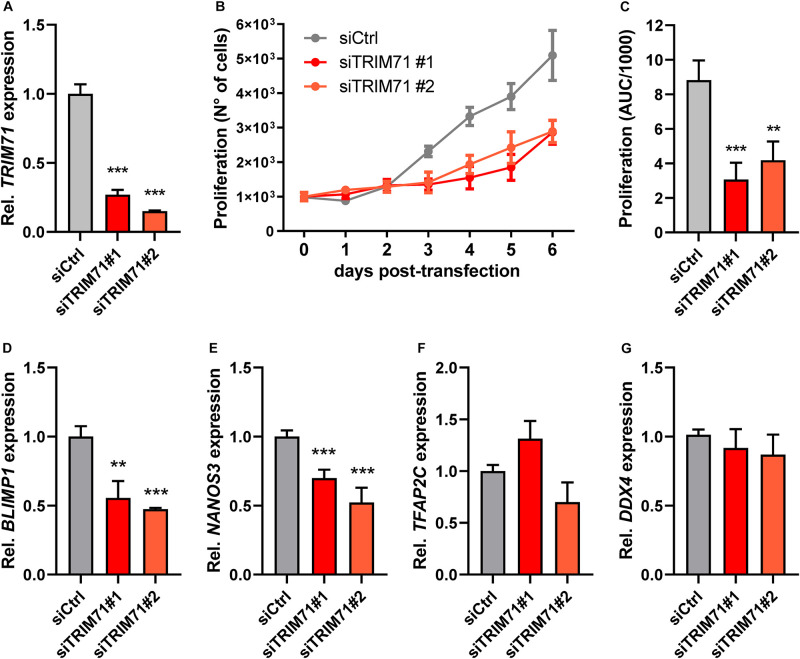
TRIM71 controls proliferation in the *in vitro* PGC-like model cell line TCam-2. **(A)** RT-qPCR measurement of *TRIM71* in wild type (siCtrl) and *TRIM71* KD (siTRIM71 #1 and siTRIM71 #2) TCam-2 cells 72 h post-transfection (*n* = 3). **(B)** Proliferation of wild type (siCtrl) and *TRIM71* KD (siTRIM71 #1 and siTRIM71 #2) TCam-2 cells depicting the increase in cell numbers over time after transfecting them with siRNAs and seeding them in equal numbers (d0) (*n* = 5). **(C)** Area under curve (AUC) measurements for the proliferation curves depicted in **B** (*n* = 5). **(D–G)** RT-qPCR measurements for the indicated PGC markers in wild type (siCtrl) and *TRIM71* knockdown (siTRIM71 #1 and siTRIM71 #2) TCam-2 cells 72 h post-transfection (*n* = 3–6). For all RT-qPCRs, the housekeeping gene *18S rRNA* was used for normalization. For all graphs, error bars represent the SEM. ***P-value < 0.005, **P-value < 0.01 (unpaired Student’s *t*-test). See also Supplementary Figure 8.

After arriving at the genital ridges, the PGC founder population strongly increases in numbers before undergoing sex determination (Tam and Snow, 1981; Anderson et al., 2000; Bowles and Koopman, 2010; Ewen and Koopman, 2010). Since TRIM71 is known to regulate proliferation in several cell lines (Chen et al., 2013; Ren et al., 2018; Hu et al., 2019; Torres-Fernández et al., 2019), we next wanted to investigate whether TRIM71 may control proliferation also in PGCs. Because the isolation of sufficient PGCs from genital ridges for its absolute reliable quantification is technically very challenging, we used the human GCT-derived seminoma cell line TCam-2, which is a widely accepted *in vitro* PGC model (Irie et al., 2015; Sugawa et al., 2015; Nettersheim et al., 2016a, b, c; Mitsunaga et al., 2017; Müller et al., 2020) and expresses high TRIM71 levels, similar to other GCT-derived cell lines (Supplementary Figure 8A). To this end, we knocked down (KD) *TRIM71* with two different siRNAs (Figure 4A), and monitored the increase in cell numbers over time (Figure 4B). Indeed, *TRIM71* KD cells proliferated significantly slower in the course of the experiment, as shown by area under curve (AUC) measurements (Figure 4C). Consistent with our previous observations in PGCLCs, *TRIM71* KD TCam-2 cells showed a significant downregulation of *BLIMP1* and *NANOS3*, while *TFAP2C* and *DDX4* remained unaltered (Figures 4D–G). Although our data do not provide evidence for a reduction in PGC proliferation *in vivo*, these results suggest that TRIM71 may be important for the expansion of the germ cell pool during embryogenesis, which could be an explanation for the reduction of male gonocytes observed in neonatal *Trim71* cKO mice.

### TRIM71 Controls Proliferation in the Germline-Derived Tumor Cell Line NCCIT

The failure of male PGCs to further differentiate into gametes can lead to the development of testicular GCT (TGCT) (Raman et al., 2005; Looijenga et al., 2010; Zorrilla and Yatsenko, 2013). TGCT can be divided into three groups: (I) tumors of newborns and infants (teratomas and yolk sac tumors), (II) tumors of adolescents and young adults (seminomas and non-seminomas) and (III) spermatocytic seminoma of elderly men (≥ 50 years) (Oosterhuis and Looijenga, 2005). TRIM71 is highly expressed in several GCT-derived cell lines (Rybak et al., 2009; Chang et al., 2012; Torres-Fernández et al., 2019) (Supplementary Figure 8A), and is upregulated in TGCT seminoma and non-seminoma patients (Tang et al., 2017; Supplementary Figures 8B–D). Since we have shown that TRIM71 controls proliferation of seminoma TCam-2 cells, we evaluated whether TRIM71 could also control proliferation in the GCT-derived non-seminoma cell line NCCIT. Because TRIM71 is strongly expressed in NCCIT cells (Supplementary Figure 8A) and we did not achieve a satisfying TRIM71 depletion via KD (data not shown), we generated *TRIM71* frameshift mutations via CRISPR/Cas9. To this end, we used two different single guide RNAs (sgRNA), one targeting the N-terminal RING domain and another targeting the C-terminal NHL domain (Supplementary Figure 8E). For the RING sgRNA (ΔRING), the generation of single NCCIT clones strikingly showed that an 83 kDa N-truncated RINGless version of TRIM71 is generated from the use of an alternative in-frame ATG codon present downstream of the targeting region (Supplementary Figure 8F). For the NHL sgRNA (ΔNHL6), generation of single clones showed that the resultant protein was either unstable or had a C-terminal truncation of the last NHL repeat (Supplementary Figure 8G), a mutation which is already known to mimic the full KO phenotype *in vivo* (Schulman et al., 2008) and to impair mRNA binding and repression *in vitro* (Torres-Fernández et al., 2019).

**FIGURE 5:**
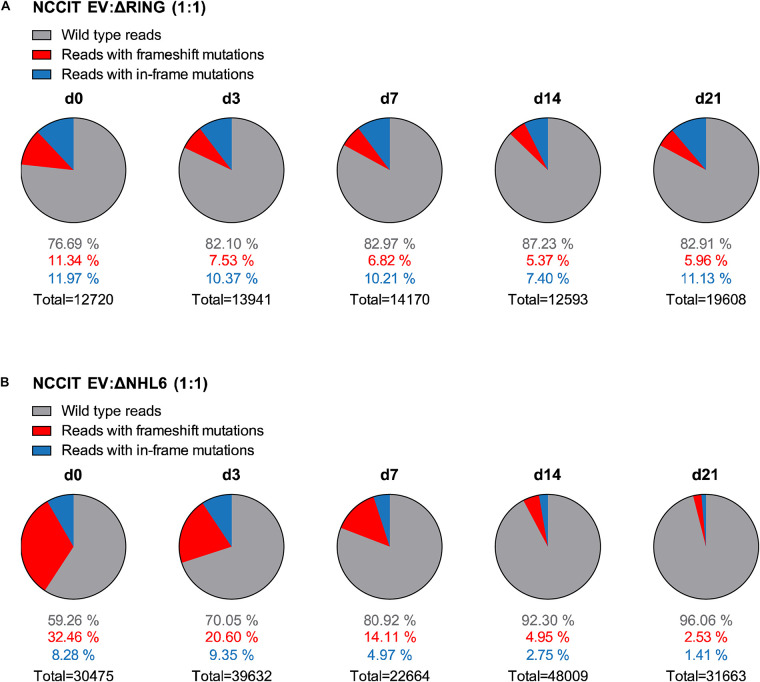
NCCIT cells with *TRIM71* mutations show population maintenance defects in growth competition assays. **(A)** Pie charts showing allele frequencies for wild type (EV) and *TRIM71* RING mutant (ΔRING) or **(B)** *TRIM71* NHL mutant (ΔNHL6) NCCIT cells obtained at different time points during growth competition assays. For the assay, NCCIT wild type cells (EV) were mixed 1:1 with either *TRIM71* RING mutant (ΔRING) or *TRIM71* NHL mutant (ΔNHL6) NCCIT cells and directly analyzed (d0) before their culturing, following by subsequent analysis at several time points (d3, d7, d14, and d21). Allele frequency was analyzed via NGS using the Illumina MiSeq platform. For *TRIM71* mutant alleles, reads with in-frame mutations and frameshift mutations (loss-of-function) in each respective domain (RING or NHL) are depicted. The total number of sequencing reads for each time point is indicated under each respective pie chart. See also Supplementary Figures 8–10.

Single cell clones often have a selection bias toward robust *in vitro* proliferation and survival. In order to study TRIM71-dependent cell proliferation in an unbiased manner, we analyzed cell population dynamics during *in vitro* growth competition assays using non-clonal mixed pools of wild type and *TRIM71* mutant cells. To this end, NCCIT cells were mock-transfected with an empty vector (EV) or with *TRIM71*-targeting vectors (ΔRING or ΔNHL6) and sorted for Cas9-GFP + cells. Bulk-transfected EV (wild type) and ΔRING/ΔNHL6 (*TRIM71* mutant) NCCIT pure populations (Supplementary Figures 9A,B) were mixed in a 1:1 ratio. We then evaluated changes in the distribution of single reads via NGS (Illumina MiSeq system) (Ravi et al., 2018) for wild type and *TRIM71* mutant alleles over a time period of 21 days and classified them as wild type reads, reads with *TRIM71* frameshift (loss-of-function) mutations and reads with *TRIM71* in-frame mutations (Figure 5). As a negative control, pure wild type NCCIT (EV) cell populations were analyzed at day 0 and day 21, showing no relevant changes in allele distribution (Supplementary Figures 9C,D). In contrast, the percentages of alleles with *TRIM71* frameshift mutations dropped ~2-fold (from 11.34% to 5.96%) for NCCIT ΔRING cells within 21 days (Figure 5A) and ~13-fold (from 32.46% to 2.53%) for NCCIT ΔNHL6 cells in the same time period (Figure 5B). Interestingly, *TRIM71* in-frame mutations also resulted in a growth disadvantage, although to a lesser degree in each case (Figures 5A,B).

In the course of these growth competition assays with NCCIT cells, we neither observed morphological changes indicative of differentiation nor signs of abnormal cell death upon regular media change, suggesting that the observed changes in population dynamics may derive from TRIM71-mediated control of proliferation. We then conducted proliferation assays on individual ΔRING and ΔNHL6 NCCIT clones for confirmation. This time, we employed the eFluor670 proliferation dye to stain NCCIT cells (d0) and measured the loss of fluorescence intensity over time as a readout for proliferation. While eFluor670 (APC) staining was comparable for wild type (EV Ctrl) and *TRIM71* mutant (ΔRING or ΔNHL6) NCCIT cells at the beginning of the experiment (d0), a retardation of eFluor670 (APC) histogram could be observed for both *TRIM71* ΔRING and ΔNHL6 NCCIT cells at the end of the experiment (d4), revealing proliferation defects in *TRIM71* mutant NCCIT cells (Supplementary Figures 10A,B). Based on the fluorescence intensity of the eFluor670 dye at d0 and d4, and assuming that fluorescence intensity halves upon every cell division, the number of cell divisions as well as the average duration of the cell cycle can be estimated (Supplementary Figures 10C,D). The cell cycles of ΔRING and ΔNHL6 mutants were 1.68 h and 3.94 h longer, respectively, than that of wild type NCCIT cells. Such a prolongation of the cell cycle corresponds to an 11.2% and 25.2% reduction in proliferation for ΔRING and ΔNHL6 mutants, respectively. The stronger effect of the ΔNHL6 mutant on proliferation may be due to the regulation of the cell cycle inhibitor *CDKN1A* (Supplementary Figure 10E), which is known to be dependent on TRIM71’s NHL domain and independent of its RING domain (Torres-Fernández et al., 2019). These results collectively demonstrate that TRIM71 is involved in the control of proliferation in NCCIT cells, and are consistent with the data obtained with TCam-2 cells as well as with previous studies involving other tumor cell lines (Chen et al., 2013; Ren et al., 2018; Hu et al., 2019; Torres-Fernández et al., 2019). Our work thereby confirms a role for TRIM71 supporting the proliferation of GCT-derived cells. Along with the elevated *TRIM71* expression observed in TGCT patients, these findings suggest that TRIM71 may not only affect germ cell proliferation during developmental processes, but may also contribute to the malignancy of GCT.

We then measured the expression of the PGC-specific markers *NANOS3* and *BLIMP1* in NCCIT wild type and *TRIM71* mutant cells. In line with our previous findings in *Trim71* KO PGCLCs and *TRIM71* KD TCam-2 cells, *NANOS3* was downregulated in *TRIM71* ΔNHL6 mutant NCCIT cells (Supplementary Figure 10F) and *BLIMP1* was downregulated in both ΔRING and ΔNHL6 mutants (Supplementary Figure 10G). Interestingly, we and others recently found that TRIM71 is not only an mRNA repressor, but is also capable of positive mRNA regulation via direct binding and stabilization (Foster et al., 2020; Torres-Fernández et al., 2021). Thus, we checked several published datasets for *Blimp1* and *Nanos3* expression and mRNA binding. In accordance with our data, *Blimp1* was found consistently downregulated in *Trim71* KO, RING mutant and NHL mutant ESCs and its mRNA was co-precipitated with TRIM71 in RNA-IPs (Supplementary Figure 10H; Welte et al., 2019) while *Nanos3* mRNA was not (data not shown). Similarly, *BLIMP1* was downregulated in *TRIM71* KO hepatocellular carcinoma (HCC) cells (Supplementary Figure 10I; Welte et al., 2019), and *BLIMP1* mRNA was also co-precipitated with TRIM71 in HCC cells (Supplementary Figure 10I; Foster et al., 2020). These results suggested a post-transcriptional regulation of *Blimp1* mRNA mediated by TRIM71, which is known to directly interact with mRNAs via conserved RNA hairpins (Kumari et al., 2018; Torres-Fernández et al., 2019; Welte et al., 2019). Supporting the RNA-IP binding data, several TRIM71-binding hairpins were found located along the *Blimp1* mRNA (Supplementary Figure 10J) while no hairpins were identified along the *Nanos3* mRNA (Welte et al., 2019). The ability of TRIM71 to positively regulate directly bound mRNAs has been reported to depend on both the RING and NHL domains, and was achieved via TRIM71-mediated repression of let-7 miRNA (Torres-Fernández et al., 2021). Interestingly, this mechanism was observed in both ESCs and HCC cells (Torres-Fernández et al., 2021). Thus, it is possible that TRIM71 regulates *Blimp1* expression via this mechanism, since (i) *Blimp1* is a known let-7 target (West et al., 2009), (ii) TRIM71 is able to directly bind *Blimp1* mRNA and (iii) TRIM71-mediated *Blimp1* regulation depends on both the RING and NHL domains.

### Exome Sequencing Data Identifies *TRIM71* Variants in Infertile Men With Severely Impaired Spermatogenesis

**FIGURE 6:**
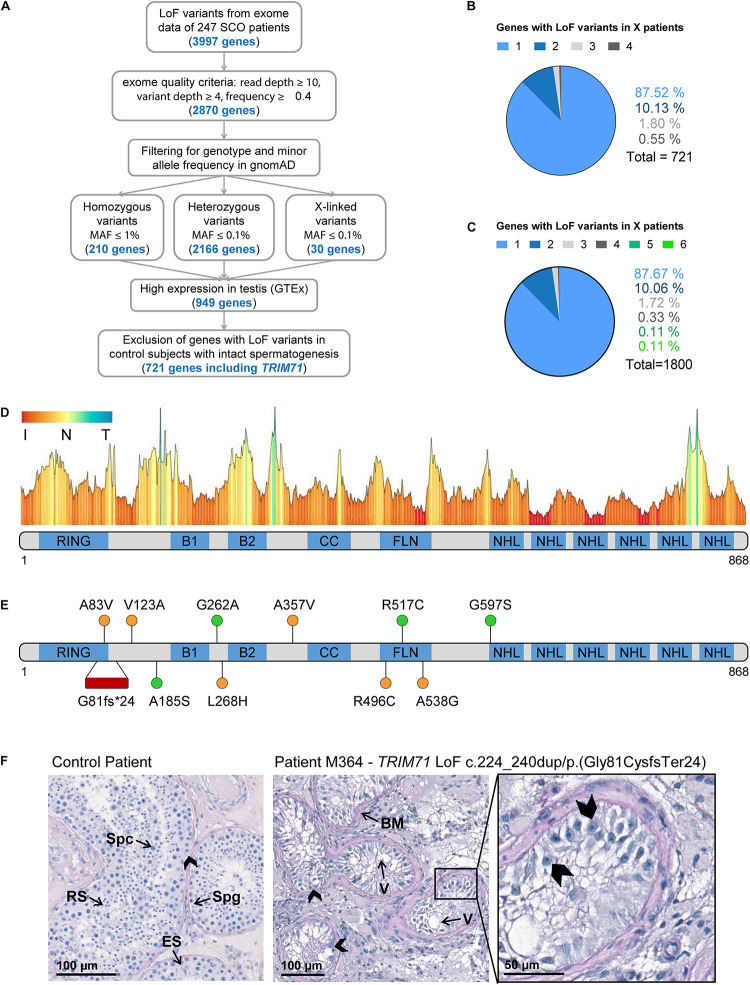
Exome sequencing in infertile SCO patients reveals an association of *TRIM71* deficiency with human male infertility. **(A)** Filtering scheme detailing gene prioritization of exome sequencing data from 247 individuals with Sertoli cell-only (SCO) phenotype using Sciobase and Haystack (see details in the section “METHODS”). Gene numbers remaining after each filtering step are indicated in blue. Loss-of-function (LoF) variants include stop, frameshift, splice acceptor and splice donor variants. gnomAD, Genome Aggregation Database; MAF, minor allele frequency; GTEx, Genotype-Tissue Expression Portal. **(B)** Percentages of genes (total = 721, obtained at the end of the filtering process depicted in **A**) affected by LoF variants in 1–4 SCO patients. None of the genes carried LoF variants in more than 4 patients in the SCO cohort (*n* = 247). **(C)** Percentages of genes (total = 1800, obtained at the end of the filtering process depicted in **A**, excluding “High expression in testis - GTEx” filtering step) affected by LoF variants in 1-6 SCO patients. None of the genes carried LoF variants in more than 6 patients in the SCO cohort (*n* = 247). **(D)** TRIM71 mutation tolerance landscape obtained from MetaDome (https://stuart.radboudumc.nl/metadome/). The graph displays missense over synonymous variant ratios per position for the entire protein, based on gnomAD variants. I, intolerant; N, neutral; T, tolerant. **(E)** Schematic representation of TRIM71 domain structural organization depicting the location of the genetic variants identified in MERGE. Red, presumably pathogenic LoF variants; orange, missense variants of uncertain significance; green, missense variants found also in proven fathers. **(F)** Periodic acid–Schiff stainings of testis sections obtained by testicular biopsy from a control patient and an SCO patient (subject M364), who harbors the *TRIM71* LoF variant c.224_240dup/p.(Gly81CysfsTer24) in heterozygosis. The control section shows intact spermatogenesis (Spg, spermatogonia; Spc, spermatocytes; RS, round spermatids; ES, elongated spermatids) as opposed to subject M364, whose histological evaluation revealed an SCO phenotype (exemplary Sertoli cells indicated by arrow heads) with several degenerated seminiferous tubules, frequently including large vacuoles (V) and thickened basement membranes (BM). See also Table 1, Supplementary Figures 11, 12, and Supplementary Tables 1, 2.

The causes of the SCO phenotype in men remain poorly understood (Tüttelmann et al., 2011; Tüttelmann et al., 2018; Koc et al., 2019). In order to identify novel associated genes, we utilized exome sequencing data of 247 SCO subjects belonging to the Male Reproductive Genomics (MERGE) study. We employed our in-house software Sciobase and our newly developed software Haystack to analyze loss-of-function (LoF) variants. After strict filtering based on quality criteria, minor allele frequency (MAF) in the general population, high expression in human testes (GTEx) and absence of LoF variants in individuals with complete spermatogenesis, we identified a total of 721 genes with LoF variants specifically found in SCO patients (Figures 6A–C). Via this independent and unbiased approach, we found *TRIM71* to be present among these genes, revealing a possible association with human male infertility. This finding is strongly supported by our previous data showing that TRIM71 deficiency results in an SCO-like phenotype in male mice. Of note, most of the identified human genes carried LoF variants in only one patient within the studied cohort and none of the identified genes carried LoF variants in more than four individuals, indicating that the SCO phenotype is highly heterogeneous (Figure 6B). Similar results were observed upon inclusion of genes with lower expression in the testis (Figure 6C).

For a further evaluation of *TRIM71* as a candidate gene associated with human male infertility, we screened 908 infertile patients - including those with SCO from MERGE - for variants in *TRIM71.* We identified 11 different rare (MAF ≤ 0.001) heterozygous *TRIM71* variants, including the aforementioned LoF variant and 10 missense variants, present in a total of 14 infertile individuals with severely impaired spermatogenesis and varying histological phenotypes (Figures 6D–F and Table 1). The majority of subjects (11 out of 14) were azoospermic, and some of them presented with reduced testicular volumes, elevated luteinizing hormone (LH) levels, elevated follicle-stimulating hormone (FSH) levels and/or reduced serum testosterone (T) levels (Table 1), all signs of broad testicular dysfunction present in 60% of azoospermic men (Tüttelmann et al., 2018).

**TABLE 1:** *TRIM71* variants identified in infertile men within the MERGE cohort.

Variant (cDNA)	Variant (protein)	Predicted effect^a^	MAF^b^	Subject ID	Age and origin	Semen analysis/histology^c^	Testis vol.^d^	LH^e^	FSH^f^	T^g^
c.224_240dup	p.(Gly81CysfsTer24)	NA	0	M364	26; Germany	Azoo/SCO	24/21	9	**17.9**	**7.6**
c.248C>T	p.(Ala83Val)	10.2; B/T/N	0	M2118	33; Germany	Crypto/NA	16/17	4.1	**7.6**	14.1
c.368T>C	p.(Val123Ala)	18.5; D/D/D	0	M2110	43; Kazakstan	Crypto/NA	19/17	5.5	6.2	14.9
c.553G>T	p.(Ala185Ser)	0.01; B/T/N	0.0047	M1325	33; Germany	Azoo/Hypo	**7/8**	4.6	6.8	15.6
c.553G>T	p.(Ala185Ser)	0.01; B/T/N	0.0047	M1562	36; Germany	Azoo/SCO	**9/6**	7.3	**24.7**	13.8
c.785G>C	p.(Gly262Ala)	0.2; B/T/D	0	M2173	28, Turkey	Azoo/SCO	**13**/18	2.9	**8.3**	17.7
c.803T>A	p.(Leu268His)	15.6; D/T/D	0	M1793	33; Turkey	Azoo/MeiA	15/15	6.3	**8.4**	15.3
c.1070C>T	p.(Ala357Val)	23.2; B/T/D	0.0001	M1686	29; Romania	Crypto/NA	17/16	9.5	**7.1**	39.7
c.1486C>T	p.(Arg496Cys)	28.8; D/D/D	0.0001	M1083	36; Germany	Azoo/NA	**12/8**	9.6	**18.1**	17.5
c.1549C>T	p.(Arg517Cys)	23.4; D/T/D	0.0016	M468	34; Pakistan	Azoo/MeiA	19/16	6.8	6.4	19.8
c.1613C>G	p.(Ala538Gly)	14.4; B/T/D	0	M2069	29; Lebanon	Azoo/NA	**9/6**	3.5	**8.4**	**6.1**
c.1789G>A	p.(Gly597Ser)	30; D/D/D	0.0022	M244	31; Germany	Azoo/MeiA	26/26	2.8	5.2	15.2
c.1789G>A	p.(Gly597Ser)	30; D/D/D	0.0022	M754	32; Turkey	Azoo/SCO	**6/7**	**11**	**30.7**	15.9
c.1789G>A	p.(Gly597Ser)	30; D/D/D	0.0022	M2141	27; Turkey	Azoo/Hypo	17/**14**	5.8	**13.5**	16.6

*^a^Predicted effect of each TRIM71 variant estimated by four different pathogenicity prediction algorithms (CADD/PolyPhen2/SIFT/MutationTaster, shown in the same order). For CADD, variants with values above 20 are more likely to be deleterious to protein function. For the rest, D, damaging/deleterious; T, tolerated; B, benign; N, neutral; NA, not available. ^b^MAF (minor allele frequency) values derive from gnomAD and are considered rare if MAF < 0.001 when occurring in heterozygosis. ^c^Semen analysis was performed for all patients (Azoo, azoospermia; Crypto, cryptozoospermia), and if biopsies were available, patients’ phenotypes were also histologically assessed (SCO, Sertoli cell-only phenotype; MeiA, meiotic arrest; Hypo, hypospermatogenesis). ^d^Testicular volumes (testis vol., right/left, ref. > 15 mL each; bold, values outside the normal range). ^e^Luteinizing hormone (LH, ref. 2-10 IU/L; bold, values outside the normal range). ^f^Follicle-stimulating hormone (FSH, ref. 1-7 IU/L; bold, values outside the normal range). ^g^Testosterone (T, ref. > 12 nmol/L; bold, values outside the normal range).*

Generally, although most of the identified missense variants were classified as variants of uncertain significance according to ACMG/AMP guidelines (Supplementary Table 1), *TRIM71* seems to be rather intolerant to missense variation (Figure 6D; Wiel et al., 2019). This was also indicated by a high Z score value of 3.28 for *TRIM71*. The Z score is a metric computed by gnomAD database and ranges from −5 to 5, with higher values indicating an intolerance to variation and, therefore, a higher likelihood for *TRIM71* variants to disrupt TRIM71 function. Furthermore, parameters such as MAF or pathogenicity prediction algorithms were used to estimate the reliability for the association of each variant with male infertility (Table 1). The conservation of the affected residues for each variant was also evaluated (Supplementary Figure 11A). Four of the *TRIM71* missense variants identified in infertile men were also found in control subjects with complete spermatogenesis (*n* = 89) or in a Dutch cohort of proven fathers (*n* = 5784), making their association with male infertility rather unlikely (Figure 6E, green). In contrast, other identified *TRIM71* missense variants were present in one patient each but neither in control subjects nor in the Dutch cohort of proven fathers (Figure 6E, orange). Of those, c.368T>C/p.(Val123Ala), c.803T>A/p.(Leu268His), c.1070C>T/p.(Ala357Val) and c.1486C>T/p.(Arg496Cys) were considered damaging by at least two out of four pathogenicity prediction algorithms (Table 1), and the respective residues for those variants were highly conserved among vertebrates (Supplementary Figure 11A). Thus, these variants have a higher likelihood of being associated with male infertility.

As an additional line of evidence, all patients carrying variants in *TRIM71* (Table 1) were evaluated for relevant variants in other genes previously reported in association with male infertility (*n* = 181, listed in Supplementary Table 2). Importantly, one heterozygous frameshift variant was found for the gene *SYCP2* in patient M1686, who carries the *TRIM71* variant c.1070C>T/p.(Ala357Val). *SYCP2* was recently associated with male infertility (Schilit et al., 2019), and it is thus unlikely that this patient’s *TRIM71* variant alone is responsible for his cryptozoospermia, but an oligogenic cause of his phenotype cannot be ruled out. Furthermore, a heterozygous missense variant for the gene *NNT* was identified in patient M1083, who carries the *TRIM71* variant c.1486C>T/p.(Arg496Cys). In this case, the *NNT* missense variant is of unclear significance according to ACMG/AMP guidelines, and its functional contribution to the patients’ phenotype can therefore neither be confirmed nor excluded at this point. Collectively, our analysis leaves the *TRIM71* variants c.368T>C/p.(Val123Ala) and c.803T>A/p.(Leu268His) as the likeliest candidates for being associated with male infertility.

### Exome Sequencing Data Reveals an Association of *TRIM71* LoF Variants With the SCO Phenotype

As mentioned above, a *TRIM71* LoF variant - c.224_240dup/p.(Gly81CysfsTer24) - was identified via exome sequencing in subject M364, who presented with a clear SCO phenotype (Figures 6E,F and Table 1). This variant consists of a duplication of 17 nucleotides which generates a frameshift resulting in a premature termination codon (PTC) downstream of the RING domain (Supplementary Figure 11B). This particular *TRIM71* LoF variant has not been described in any public database so far and was classified as likely pathogenic according to ACMG/AMP guidelines (Supplementary Table 1). Furthermore, the observed/expected (o/e) score computed for *TRIM71* in the gnomAD database was extremely low (o/e score = 0.04, 90% confidence interval 0.01–0.17). The o/e score compares the number of observed versus theoretically expected LoF variants for a gene of interest. Without any selection pressure applied, an o/e ratio of around 1 would be expected for any given gene, whereas values below 0.35 are a rather clear sign of selection pressure against LoF variants, likely leading to haploinsufficiency intolerance. Furthermore, subject M364 carries neither additional LoF variants nor likely pathogenic missense variants in any other known infertility-associated genes (listed in Supplementary Table 2). This patient presented with normal testicular volumes and LH levels, but increased FSH and decreased testosterone levels (Table 1), as is often the case in men with impaired spermatogenesis. Of note, no *TRIM71* LoF variants were found in the Dutch cohort of proven fathers, and only one LoF variant was present in the exome data from 125,748 presumably healthy individuals from gnomAD (Karczewski et al., 2020). This constitutes a significant enrichment of *TRIM71* LoF variants in the MERGE cohort (*n* = 908; *p* = 0.01) and an even higher enrichment in the SCO subcohort (*n* = 247; *p* = 0.004), supporting a reliable association of *TRIM71* LoF variants with human male infertility. Thus, the reported *TRIM71* LoF variant is likely the cause of subject M364’s SCO phenotype, underscoring a haploinsufficiency of *TRIM71* in human spermatogenesis. In contrast, although sperm counts were strongly reduced in *TRIM71* heterozygous mice, these animals were fertile. This discrepancy may be attributed to inherent mechanistic differences between murine and human gametogenesis. Indeed, it has been reported for multiple diseases that the modulation of a specific gene can result in different severity and range of symptoms for mice and humans (Elsea and Lucas, 2002).

In order to address the functionality of all identified *TRIM71* variants, we overexpressed them in HEK293T cells to evaluate protein stability via western blot analysis. Whereas all *TRIM71* missense variants yield stable proteins, the LoF variant resulted in a functional KO as expected, since no protein was detected via western blot analysis for the respective LoF construct Flag-dupPTC (Supplementary Figure 12A). We additionally tested the ability of the different TRIM71 constructs to repress the 3′UTR of *CDKN1A* (Torres-Fernández et al., 2019) as well as the activity of let-7 miRNA (Torres-Fernández et al., 2021) via luciferase reporter assays in HEK293T cells. *CDKN1A* 3′UTR repression was impaired for the Flag-dupPTC construct, and significantly diminished for the G597S variant which affects TRIM71’s NHL domain (Supplementary Figures 12B,C). The ability of TRIM71 to derepress a luciferase reporter under the control of a 3′UTR with multiple let-7 binding sites was also abrogated for the Flag-dupPTC construct (Supplementary Figures 12D,E). These assays collectively show that the *TRIM71* LoF variant identified in the patient with SCO phenotype yields a functional KO. Future experiments should determine whether the here identified missense variants impair other known TRIM71 functions (e.g., TRIM71’s E3 ubiquitin ligase role) as well as determine their pathogenicity in the context of human infertility.

## Discussion

TRIM71 is a stem cell-specific protein expressed early in development and with an essential function for embryogenesis (Schulman et al., 2005, 2008; Rybak et al., 2009; Cuevas et al., 2015). Embryonic lethality in *Trim71* full KO mice is accompanied by neural tube closure defects, revealing also a function of TRIM71 in the development of the nervous system (Schulman et al., 2005, 2008; Rybak et al., 2009; Cuevas et al., 2015). Indeed, TRIM71 is known to promote self-renewal of neural progenitor cells (Chen et al., 2012), and *TRIM71* variants affecting its NHL domain have been associated with human congenital hydrocephalus, a brain developmental disease characterized by enlarged brain ventricles due to an abnormal accumulation of cerebrospinal fluid (Furey et al., 2018). Furthermore, TRIM71 has been connected with carcinogenesis in several studies (Chen et al., 2013, 2019; Ren et al., 2018; Hu et al., 2019; Torres-Fernández et al., 2019). Altogether, previous research on TRIM71 has been mostly focused on embryonic and neural stem cells as well as cancer cells. Our work here reports a novel role for TRIM71 in the development of germ cells during embryogenesis with crucial implications in murine and possibly human fertility.

In line with previous studies (Rybak et al., 2009; Du et al., 2020), our work reveals an expression of *Trim71* in adult mouse testes which we found to be confined to SSCs in mice and human. We showed that germline-specific ablation of *Trim71* early in mouse development causes a substantial reduction of gonad size and infertility in both sexes. Further characterization of adult *Trim71* cKO mouse testes revealed a significant downregulation of SSC-specific and spermatid markers caused by a strong reduction in the number of developing germ cells, a condition which in humans is known as Sertoli cell-only (SCO) phenotype (Tüttelmann et al., 2011, 2018; Koc et al., 2019). Via exome sequencing of human infertile men, we uncovered an association of *TRIM71* deficiency with the SCO phenotype.

In humans, infertility affects 10–15% of couples trying to conceive (Zorrilla and Yatsenko, 2013). Male factors contribute in about half of couples and usually genetic causes correlate with a more severe spermatogenic impairment (Lee et al., 2011). Lately, several genes have been identified as monogenic causes for azoospermia due to meiotic arrest (e.g., TEX11 Yatsenko et al., 2015, STAG3 Van Der Bijl et al., 2019, M1AP Wyrwoll et al., 2020 and SHOC1 Krausz et al., 2020). However, the SCO phenotype seems to be a highly heterogeneous condition, as indicated by our data, making the identification of monogenic causes especially challenging. Our work uncovers *TRIM71* as the first monogenic cause for this condition, based on the SCO-like phenotype observed in our germline-specific *Trim71* cKO mouse together with the *TRIM71* LoF variant that we found in an SCO patient. The finding of *TRIM71* as a novel SCO candidate gene is relevant in terms of patient care, as it may allow future patients to be provided with a causal diagnosis for their azoospermia, and the potential success of testicular biopsy and sperm extraction with the aim to perform *in vitro* fertilization could be predicted in advance.

In contrast to the convincing relevance of the *TRIM71* LoF variant, assessing the role of the identified rare *TRIM71* missense variants in infertile patients of varying histological phenotypes is much more challenging. Of note, variants in the same gene may cause a spectrum of histological phenotypes (Wyrwoll et al., 2020). Although our analysis provides a prediction for the pathogenicity of these variants, their functional effects on human male fertility are yet of unclear significance and can be only determined experimentally. Nevertheless, it is worth emphasizing that most missense variants identified in *TRIM71* cluster outside of the NHL domain, as mutations affecting the NHL domain have been proven to be highly deleterious (Slack et al., 2000; Kumari et al., 2018) and compromise survival during early embryogenesis (Schulman et al., 2008). Accordingly, we found that these missense variants do not compromise NHL-dependent TRIM71 functions. Future experiments using these *TRIM71* variants should determine whether they functionally affect TRIM71’s E3 ubiquitin ligase role, and if so, how exactly TRIM71-mediated ubiquitylation contributes to germ cell development.

Our germline-specific *Trim71* cKO mouse model provided further evidence for the role of *Trim71* in germ cell development. The SCO-like phenotype was already apparent in the testes of neonatal (P0.5) cKO mice, indicating that TRIM71-induced germline defects have an embryonic origin. However, early developmental defects are likely amplified during postnatal mitotic reactivation and pubertal spermatogenesis as described by a recent work showing infertility in a different germline-specific *Trim71* knockout (*Trim71*^–/fl^*; Ddx4*^*Cre/*+^) mouse model (Du et al., 2020). In contrast to our observations in *Trim71*^*fl/*–^*; Nanos3*^*Cre/*+^ mice, defects in *Trim71*^–/fl^*; Ddx4*^*Cre/*+^ mice were only detected in pubertal (P10) and adult (P56) mice, with germ cell numbers not yet altered in neonatal (P1) mice (Du et al., 2020). This discrepancy may result from an earlier Cre recombinase expression in our cKO model, since *Nanos3* is expressed during PGC specification (E7.5) (Ginsburg et al., 1990; Lawson et al., 1999; Tsuda et al., 2003), and *Ddx4* is expressed after colonization of the genital ridges (E10.5) (Fujiwara et al., 1994; Tanaka et al., 2000). In fact, *Ddx4*-induced recombination was previously reported to occur even after sex determination as late as E15.0 (Gallardo et al., 2007). This might also explain why *Trim71*^*fl/*–^*; Ddx4*^*Cre/*+^ females are fertile (Prof. Xin Wu, personal communication, June 22, 2020), while we found *Trim71*^*fl/*–^*; Nanos3*^*Cre/*+^ females to be sterile. Notably, studies in *C. elegans* showed that the nematode homolog of TRIM71, LIN-41, is required for normal oocyte growth and meiotic maturation, with *lin-41* depletion causing sterility in females (Spike et al., 2014; Tsukamoto et al., 2017). Further studies are required to characterize the function of TRIM71 in mammalian female gonad development and fertility.

Although we do not exclude sex-specific functions of TRIM71 in the germline, we found both male and female *Trim71* cKO mice to be infertile, suggesting that germ cell developmental defects may occur prior to sex determination of PGCs. Alternatively, TRIM71 deficiency may similarly affect male and female germ cells after sex determination, since published datasets revealed similar TRIM71 expression patterns in male and female PGCs during development. Via *in vitro* differentiation of ESCs into PGCLCs, we observed reduced numbers of *Trim71*-deficient PGCLCs at the end of the specification process. Although such a reduction in the PGCLC numbers would underscore mild PGC specification defects of *Trim71*-deficient cells, it could also result from a specific downregulation of the PGC marker SSEA-1/CD15 which was used for PGCLC staining, and which was found downregulated in *Trim71* KO PGCLCs. Furthermore, *Trim71*-deficient PGCLCs showed a downregulation of *Blimp1* and *Nanos3*, which are two well-known master regulators of PGC development (Kurimoto et al., 2008; Suzuki et al., 2008). Consistently, we also found reduced levels of *BLIMP1* and *NANOS3* in *TRIM71* KD TCam-2 cells and *TRIM71* mutant NCCIT cells. We furthermore presented evidence suggesting that TRIM71-mediated *BLIMP1* regulation may be attributed to a recently reported novel role for TRIM71 in the repression of let-7 miRNA activity (Torres-Fernández et al., 2021). In this context, it is worth mentioning that the let-7-mediated regulation of *Blimp1* plays an important role in murine PGC development (West et al., 2009). Interestingly, *Trim71*-deficient ESCs showed a premature upregulation of differentiation-promoting miRNAs, including let-7 and several gonad-specific miRNAs (Mitschka et al., 2015; Torres-Fernández et al., 2021), highlighting a yet-unknown role for TRIM71-mediated miRNA regulation in early gonad development. It is however intriguing that TRIM71-mediated *Blimp1* regulation does not result in PGC specification or migration defects, as BLIMP1 is well known to control early PGC functions (Ohinata et al., 2005). At this point, we can only speculate that such a moderate regulation of *Blimp1* at early stages may induce, for instance, epigenetic changes that may affect later stages of germ cell development. Alternatively, TRIM71-mediated *Blimp1* regulation may simply be more relevant at later stages, as the expression of both TRIM71 and BLIMP1 is indeed sustained in developing germ cells even after sex determination (Seisenberger et al., 2012; Zhao et al., 2018).

Since *Trim71* expression was still remarkably high in PGCLCs, we aimed at investigating further roles of TRIM71 downstream of PGC specification. While we excluded *in vivo* PGC migration defects upon *TRIM71* depletion, *in vitro* proliferation assays with TCam-2 and NCCIT cells showed that TRIM71 actively promotes proliferation of these GCT-derived cell lines. Given the similarities of TCam-2 cells – and to a lesser extent of NCCIT cells – with PGCs (Irie et al., 2015; Sugawa et al., 2015; Nettersheim et al., 2016a, b, c; Mitsunaga et al., 2017; Müller et al., 2020), our results suggest that TRIM71 may be involved in the control of PGC proliferation after they reach the genital ridges, when a significant expansion of the PGC pool occurs (Tam and Snow, 1981; Anderson et al., 2000; Bowles and Koopman, 2010; Ewen and Koopman, 2010). To strengthen this notion, future experiments should investigate the role of TRIM71 in the proliferation of post-migratory PGCs *in vivo*. Additionally, the role of TRIM71 in the maintenance of the germline may extend beyond proliferation control, as previous studies have shown roles for TRIM71 not only in the control of proliferation (Chang et al., 2012; Chen et al., 2012, 2013; Ren et al., 2018; Hu et al., 2019; Torres-Fernández et al., 2019), but also during differentiation (Loedige et al., 2013; Worringer et al., 2014; Mitschka et al., 2015) and apoptosis (Nguyen et al., 2017; Chen et al., 2019; Hu et al., 2019) in developmental and oncogenic contexts. Importantly, a recent study described an impaired proliferation and an increased apoptosis in adult mice primary SSCs upon *Trim71* KD (Du et al., 2020). Likewise, TRIM71 might also participate in the control of the massive apoptotic waves that mitotically arrested male PGCs/gonocytes undergo within the developing and neonatal gonads (Wang et al., 1998).

In summary, we have shown that germline-specific *Trim71* cKO mice are infertile, with males predominantly displaying an SCO-like phenotype. We have identified *TRIM71* variants in infertile men with severely impaired spermatogenesis, including a LoF variant in an SCO patient which was confirmed to abrogate TRIM71 expression and function. *Trim71*-associated infertility seems to originate during embryonic development, since the SCO-like phenotype was already apparent in neonatal P0.5 *Trim71* cKO male mice and *Trim71* expression in females is restricted to fetal gonads. Our *in vitro* assays consistently showed TRIM71-dependent changes in cell proliferation as well as in the expression of important PGC regulatory factors, suggesting that infertility in *Trim71* cKO mice may be caused by an impaired proliferation of germ cells. Altogether, our work supports a novel role for TRIM71 in the embryonic development of the germline as well as of germline-derived tumors or GCT. Future unraveling of the molecular mechanisms by which TRIM71 governs germ cell biology will shed further light on the underlying causes of infertility. A deeper understanding of these processes will contribute to the development of new diagnostic and therapeutic strategies for reproductive medicine and for the treatment of GCT.

## Methods

### Mouse Generation

All animal experiments were conducted in a licensed animal facility in accordance with the German law on the protection of experimental animals (the German animal welfare act), and were approved (approval number 87-51.04.2011.A063) by local authorities of the state of Nordrhein-Westfalen (Landesamt für Natur-, Umwelt- und Verbraucherschutz NRW).

The generation of the full *Trim71* KO mouse (*Trim71*^*fl/fl*^*; Rosa26-CreERT2*) was previously described (Mitschka et al., 2015). In order to generate a germline-specific *Trim71* knockout mouse, wild type females with floxed *Trim71* alleles (WT, *Trim71*^*fl/fl*^) were bred with male mice expressing the Cre recombinase under the control of the endogenous *Nanos3* promoter in heterozygosity (*Nanos3*^*Cre/*+^) (Suzuki et al., 2008). The heterozygous *Trim71*^–/+^*; Nanos3*^*Cre/*+^ male offspring was then crossed with wild type *Trim71*^+/+^ females to produce *Trim71* wild type animals with the *Nanos3*-Cre allele in heterozygosity (WT, *Trim71*^+/+^*; Nanos3*^*Cre/*+^), or with wild type *Trim71*^*fl/fl*^ females to produce germline-specific heterozygous (cHET, *Trim71*^–/+^*; Nanos3*^*Cre/*+^) and germline-specific knockout (cKO, *Trim71*^–/fl^*; Nanos3*^*Cre/*+^) animals.

### Genomic DNA Extraction and Genotyping

Genomic DNA was extracted from adult mice tail or ear biopsies or from mouse embryo yolk sacs by boiling the tissue in 50 mM NaOH for 20 min followed by pH neutralization of the lysate with 1/4 of 1 M Tris-Cl, pH 8.0. A three-primer strategy was used for PCR amplification of the *Trim71* locus or the *Nanos3* locus and fragments were resolved on a 2% agarose gel. Primers used are listed in Supplementary Table 3.

### Epidydmal Sperm Counts

Cauda epididymis were isolated from 3-month-old male mice and transferred into 0.5–1 ml of PBS pre-warmed at 35–37°C. Sperm release was achieved by performing multiple cauda incisions followed by 10-min incubation at 35–37°C to allow the sperm swim out. The sperm suspension was then diluted in water (1:15-1:40) and sperm count was determined using a Neubauer hemocytometer.

### Isolation of Spermatogonial Stem Cells From Mouse Testes

Testes were isolated from 2-month-old male mice, and seminiferous tubules were exposed by removing the tunica albuginea. The tubules of several testes were pooled and incubated in approximately 10 volumes of HBSS with calcium and magnesium containing 1 mg/ml collagenase Type IV and 200 to 500 μg/ml DNAse I, followed by incubation at 37°C under gentle agitation for 15 min. The tubules were then washed three times in 10 volumes of HBSS, followed by incubation at 37°C for 5 min in HBSS containing 0.25% trypsin and 150 μg/ml DNAse I under gentle agitation. Trypsinization was stopped by adding 20% FBS, and the cell suspension was filtered through a 40 μm pore size nylon filter. The filtrate was centrifuged at 1000 rpm for 5 min at 4°C and the cell pellet was resuspended in PBS before cell counting. Magnetic-activated cell sorting (MACS) was used to enrich THY1.2 + cells. To this end, 10^6^ cells were stained with 0.2 μg FITC-labeled anti-THY1.2/CD90.2 antibody (BioLegend) for 15 min at 4°C. The cells were then washed in MACS-buffer (PBS supplemented with 2 mM EDTA and 0.5% FBS) and incubated in a 1:5 dilution of anti-FITC microbeads (Miltenyi) for 15 min at 4°C before a final washing in MACS buffer followed by centrifugation at 1000 rpm for 5 min at 4°C. The pellet was resuspended in 500 μl MACS buffer and loaded on an AutoMACS device (Miltenyi) with the program set for positive selection. The efficiency of enrichment was 8-fold as later controlled by flow cytometry. The different cell populations were then used for RNA extraction and qRT-PCR quantification.

### H&E Staining of Mouse Testes Cross-Sections

PFA-fixed paraffin-embedded murine testes cross-sections were deparaffinized by incubation at 65°C for 15-20 min until the paraffin wax had melted, and washed in xylol twice for 10 min. Next, the sections were rehydrated by a descending ethanol dilution series (100% (2x), 95%, 90%, 80%, 70%) for 30 sec each and were kept in distilled water until staining. Cross-sections were stained in haematoxylin for 3 min and washed in cold running tap water for 3-5 min. Afterward, the sections were counterstained with 0.5% eosin for 3 min, removing excess dye by rinsing in cold running tap water for 1 min. Cross-sections were then dehydrated in an ascending ethanol dilution series [70%, 80%, 90%, 95%, 100% (2x)] and cleared in xylol twice for 2 min. Paraffin sections were then mounted with the xylene-based DPX mounting media for histology (Sigma-Aldrich). H&E stained sections were stored under the fume hood for 24 h before imaging by bright-field microscopy using the Zeiss Axio Lab.A1 microscope (Carl Zeiss).

### Immunofluorescence Staining of Testes Cryosections

PFA-fixed murine testis cryosections stored at −20°C were defrosted and dried at room temperature for 15 min before their rehydration by washing in PBS and rinsing in distilled water. After drying the slides at RT for 10–15 min, cryosections were blocked (PBS supplemented with 1% BSA, 2% donkey serum and 0.3% Triton X-100) for 1 h at RT while covered with parafilm to prevent evaporation. Primary antibodies diluted in PBST (0.3% Triton X-100/PBS) supplemented with 1% BSA were added and incubated at 4°C overnight. On the next day, sections were washed three times for 5 min with PBST, and fluorescently conjugated secondary antibodies diluted in PBST supplemented with 1% BSA were added and incubated in the dark for 1 h at RT. The sections were then washed three times for 5 min with PBST and slides were mounted with Fluoromount-G containing DAPI and photobleaching inhibitors (SouthernBiotech). All steps were performed in a dark humid chamber to prevent evaporation and photobleaching. Stained sections were stored at 4°C for 24 h prior to imaging by immunofluorescence microscopy. Images were taken using the Zeiss Observer.Z1 epifluorescence microscope (Carl Zeiss) and the ZEN 2012 (blue edition) software (Carl Zeiss). Antibodies used are listed in Supplementary Table 4.

### RNA Extraction and qRT-PCR Quantification

RNA was extracted from cell pellets using the Trizol-containing reagent peqGold TriFAST according to the manufacturer’s instructions (PeqLab). RNA pellets were resuspended in RNase-free water, and DNA digestion was performed prior to RNA quantification. 0.5–1 μg of RNA was reverse transcribed to cDNA using the High Capacity cDNA Reverse Transcription Kit (Applied Biosystems) according to the manufacturer’s instructions. The cDNA was then diluted 1:5, and a relative quantification of specific genes was performed in a Bio-Rad qCycler using either TaqMan probes in iTaq Universal Probes Supermix or specific primer pairs in iTaq Universal SYBR Green Supermix (BioRad). Probes and Primers used are listed in Supplementary Table 3.

### Protein Extraction and Western Blotting

Cell pellets were lyzed in RIPA buffer (20 mM Tris–HCl pH 7.5, 150 mM NaCl, 1 mM Na_2_EDTA, 1 mM EGTA, 1% NP-40, 1 mM Na_3_VO_4_, 1% sodium deoxycholate, 2.5 mM sodium pyrophosphate, 1 mM glycerophosphate) supplemented with protease inhibitors and protein lysates were pre-cleared by centrifugation and quantified using the BCA assay kit (Pierce) according to the manufacturer’s instructions. Protein lysates were then denatured by incubation with SDS buffer (12% glycerol, 60 mM Na_2_EDTA pH 8, 0.6% SDS, 0.003% bromophenol blue) for 10 min at 95°C and separated in SDS-PAGE gels in Laemmli buffer (25 mM Tris, 192 mM glycine, 0.1% SDS). Proteins were then wet transferred to a nitrocellulose membrane in transfer buffer (25 mM Tris–HCl pH 7.6, 192 mM glycine, 20% methanol, 0.03% SDS) and membranes were blocked with 5% milk powder (w/v) diluted in 1 × TBST (50 mM Tris–HCl pH 7.6, 150 mM NaCl, 0.05% Tween-20) prior to overnight incubation at 4°C with the required primary antibodies. After washing the membrane three times with 1 × TBST, they were incubated with a suitable HRP-coupled secondary antibody for 1 h at RT, followed by three washing steps with 1 × TBST. Membranes were developed with the ECL substrate kit (Pierce) according to the manufacturer’s instructions. Antibodies used are listed in Supplementary Table 4.

### Cell Culture and Transfections

Derivation of wild type murine ESCs (WT, *Trim71*^*fl/fl*^) from conditional *Trim71* full KO mice (*Trim71*^*fl/fl*^*; Rosa26-CreERT2*) was previously described (Mitschka et al., 2015). *Trim71* knockout murine ESCs (KO, *Trim71*^–/–^) were generated from wild type ESCs by addition of 500 nM of 4-hydroxytamoxifen in their culture media for 48 h, followed by further culture for 72 h to achieve full protein depletion. ESCs were cultured in 0.1% gelatin-coated dishes and maintained in 2i + LIF DMEM media (DMEM knockout media supplemented with 15% FCS, 1% penicillin-streptomycin, 0.1 mM NEAA, 2 mM L-GlutaMAX, 100 μM β-mercaptoethanol, 0.2% in-house produced LIF, 1 μM of MEK/ERK inhibitor PD0325091 and 3 μM of GSK-3 inhibitor CHIR99021).

The human hepatocellular carcinoma cell line HepG2 and the human embryonic carcinoma cell lines JKT-1, NCCIT, NTERA-2, TCam-2 and 2102EP were acquired from ATCC. HepG2, NCCIT and TCam-2 were cultured in RPMI 1640 media supplemented with 10% FBS and 1% penicillin–streptomycin antibiotic solution. JKT-1, NTERA -2 and 2012EP cells were cultured in DMEM media supplemented with 10% FBS and 1% penicillin-streptomycin antibiotic solution. DNA transfection in HEK293T cells was conducted with Lipofectamine2000 reagent following the manufacturer’s instructions and using 1 μg DNA per μl of Lipofectamine. RNA transfection in TCam-2 cells was performed with Lipofectamine RNAiMAX reagent following the manufacturer’s instructions and using 10 pmol siRNA per 2 μL of Lipofectamine. For proliferation assays in TCam-2 cells, 10^3^ cells per well were seeded, transfected with the siRNAs in suspension (d0), and cell numbers were monitored over the course of several days (d1-d6).

### *In vitro* Differentiation of ESCs Into PGCLCs

Embryonic stem cells growing under naïve pluripotency (d0) conditions on 0.1% gelatine-coated dishes and 2i + LIF N2B27 media were primed to EpiLCs (resembling post-implantation EpiSCs) for two days by plating 10^5^ cells per well on 12-well plates coated with 20 μg/ml fibronectin in priming media (N2B27 media supplemented with 1% penicillin-streptomycin, 2 mM L-GlutaMAX, 1% KSR, 20 ng/ml Activin A and 12 ng/ml bFGF). After 24 h (d1), fresh priming media was provided. After 48 h (d2), EpiLCs were detached with Accutase Stem Cell Pro and 5000 cells per well were plated on a suspension U-bottom 96-well dish in specification media (GMEM media supplemented with 15% KSR, 1% penicillin-streptomycin, 0.1 mM NEAA, 2 mM L-GlutaMAX, 100 μM β-mercaptoethanol, 1 mM sodium pyruvate, 0.2% in-house produced LIF, 50 ng/ml EGF, 100 ng/ml SCF and 500 ng/ml BMP4). After 6 days in this media (d8), cells growing as spheroids were recovered, detached by trypsinization for 10 min at 37°C and either used for RNA extraction or double-stained with anti-ITGB3/CD61 and anti-SSEA-1/CD15 antibodies to determine the number of PGCLCs by flow cytometry. For staining, trypsinized cells were washed in 0.1% BSA/PBS, incubated with the antibodies diluted in 0.1% BSA/PBS for 15 min at 4°C in the dark and washed again in 0.1% BSA/PBS. Isotype control antibodies were used to control single stainings (data not shown). These protocols were adapted from a previous work (Hayashi et al., 2011). Antibodies used are listed in Supplementary Table 4.

### Analysis of *in vivo* PGC Migration

E8.5-8.75 embryos were obtained from pregnant females with the first day of vaginal plug identification defined as 0.5 dpc. Embryos were dissected in PBS and the upper body was cut off and used for genotyping. The lower body was dissected to expose the hindgut and fixed in 4% PFA for 3 h at 4°C, followed by several washings in PBS. Embryos were then placed in 70% ethanol at 4°C for 1 h, washed once in PBS and stained with Fast Red TR and α-naphthyl phosphate (Sigma) following the manufacturer’s instructions to detect alkaline phosphatase-positive cells.

### Generation of NCCIT Cells With *TRIM71* Frameshift Mutations

Gene editing using the CRISPR/Cas9 system was carried out using the plasmid pSpCas9(BB)-2A-GFP (PX458), which was a kind gift from Feng Zhang (Addgene plasmid #48138), after the insertion of specific sgRNAs targeting TRIM71 RING (sgRNA ΔRING 5′-CACCGCTCGCAGACGCTCACGCTGT-3′) or NHL (sgRNA ΔNHL6 5′-CACCGCACAACGATCATTCCGCTGG-3′) domains. NCCIT cells were transfected with PX458 empty vector (EV), TRIM71 ΔRING vector or TRIM71 ΔNHL6 vector using Lipofectamine Stem Transfection Reagent (Invitrogen) following the manufacturer’s instructions. 48 h post- transfection, transfected (GFP-positive) cells were sorted by FACS and plated as a bulk population. Growth competition assays (see below) were performed with sorted bulk populations. Generation of single clones from the bulk populations was also performed for subsequent Sanger sequencing, western blot analysis, RNA extraction and qPCR measurements, and eFluo670 proliferation assays (see below).

### Growth Competition Assays and NGS Allele Frequency Analysis via Illumina MiSeq

In order to analyze the growth behavior of CRISPR/Cas9-edited NCCIT cells, GFP-positive sorted bulk EV-transfected cells (WT) were mixed 1:1 with either *TRIM71* sgRNA ΔRING-transfected cells or *TRIM71* sgRNA ΔNHL6-transfected cells (d0) and cultured (2 × 10^5^ cells per well in a 6-well plate) for three weeks to investigate the population dynamics. Samples (2 × 10^5^ cells) were taken at d0, d3, d7, d14 and d21 for genomic DNA extraction. A double PCR strategy was then applied for the preparation of barcoded amplicons. In a first PCR reaction, the region around the CRISPR/Cas9-targeted site was amplified using site-specific forward and reverse primers with a common 5′ overhang. After PCR product cleaning, a second PCR was conducted with Illumina index primers which bind to the common 5′overhangs of the first primers and also contain unique barcode/index sequences followed by specific 5′ (P5) or 3′ (P7) adaptor sequences. PCR products were then gel-purified and sequenced on an Illumina MiSeq sequencing platform via amplification with common primers binding to P5/P7 adaptor sequences. Data analysis was performed using CRISPResso2 (Pinello et al., 2016) with the quality cut-off set at 30 and the minimum identity score for the alignment being adjusted to 50. After analysis of the sequencing data, single reads were categorized as wild type, frameshift mutations and in-frame mutations, and allele frequencies were calculated and displayed as percentages in a pie chart. Comparing changes in the distribution of single reads for wild type (EV) and frameshift mutants TRIM71 ΔRING or TRIM71 ΔNHL6 over time gives insights into the dynamics of each cell populations within the initially 1:1 mixed culture. Allele frequencies of pure wild type (EV) were also analyzed at d0 and d21 as a control. Analysis of allele frequencies of pure WT, TRIM71 ΔRING and TRIM71 ΔNHL6 populations before (d0 pure) and after mixing them 1:1 (d0 1:1) ensured that no PCR product was biased over another based on theoretically expected *versus* observed allele frequencies.

### eFluor670 Proliferation Assays in NCCIT Cells

Cells were stained with the proliferation dye eFluor670 (eBiosciences) diluted to 5 μM in PBS (1 ml of PBS per 10^6^ cells) for 10 min at 37°C in the dark. The labeling was stopped by addition of 4-5 volumes of cold FCS and incubation for 5 min on ice. Cells were washed once with complete growth medium and once with PBS before seeding them at different densities per condition in multi-well plates. A fraction of the cells was used to measure the initial fluorescence intensity (day 0) via flow cytometry. The loss of fluorescence intensity overtime was monitored by flow cytometry for 4 days after staining. Flow cytometry data was then analyzed with FlowJo. The number of cell divisions at the end of the experiment was calculated assuming a decrease of the median fluorescence intensity (MFI) by half upon each cell division with the following formula: Division Number = log_2_ [(MFI_*day*__0_-MFI_*unstained*_)/(MFI_*day*__4_-MFI_*unstained*_)]. The average cell cycle duration was estimated at the end of the experiment from the number of cell divisions.

### Exome Sequencing Study Population

The study population consisted of 1025 individuals (MERGE cohort), of whom 908 otherwise healthy men presented with quantitative spermatogenic impairment. Patients attended the Centre of Reproductive Medicine and Andrology (CeRA) at the University Hospital Münster or the Clinic for Urology, Pediatric Urology and Andrology in Gießen. Neither individuals with previous chemo-/radiotherapy history nor patients with Klinefelter syndrome or AZF deletions, which represent known genetic causes of male infertility, were included in this study. A subgroup of 247 selected patients was used to investigate novel genetic causes of the SCO phenotype (SCO subcohort). They had previously undergone testicular biopsy and presented with complete and bilateral absence of germ cells. Specifically, two biopsies were taken from each testis, and tissue sections were done from each biopsy followed by the evaluation of all tubules in one random section per biopsy for exact determination of the phenotype. In addition to this subgroup, 89 male subjects with complete spermatogenesis also pertaining to the MERGE cohort were included in the study as controls. All individuals gave written informed consent and the study protocol has been approved by the appropriate ethics committees according to the Declaration of Helsinki (Münster: Kennzeichen 2010-578-f-S, Gießen: No. 26/11). As further controls, we utilized a Dutch cohort of 5784 proven fathers sequenced at the Radboudumc genome diagnostics center in Nijmegen, the Netherlands. These were healthy fathers of a child with severe developmental delay and all parents underwent routine exome sequencing for trio analysis. The fertility of the fathers is expected to be similar to an unselected sample of the population.

### Exome Sequencing Data Generation and Analysis

In order to perform exome sequencing, genomic DNA was isolated from the subjects’ peripheral blood applying standard procedures. Target enrichment, library capture, exome sequencing and variant calling were performed as previously described (Wyrwoll et al., 2020). Briefly, DNA was enriched according to Agilent’s SureSelect^*QXT*^ Target Enrichment for Illumina Multiplexed Sequencing Featuring Transposase-Based Library Prep Technology or Twist Bioscience’s Twist Human Core Exome protocol. For library capture, SureSelectXT Human All Exon Kits (Agilent) or Human Core Exome (Twist Bioscience) were used. Sequencing was then conducted on Illumina HiScanSQ^®^, Illumina NextSeq^®^500/550 or Illumina HiSeqX^®^ systems.

Exome data analysis was conducted on 247 SCO patients using our platform Sciobase. We filtered genes based on the predicted effect of the variant on translated protein (including only stop-gain, frameshift, and splice donor/acceptor variants) and the allele frequency in the general population (including only rare variants (MAF ≤ 0.01) as listed in gnomAD genomic database (v2.1.1^1^). This first filtering step resulted in the selection of 3997 genes. We then developed an application termed Haystack to facilitate the search for previously unknown causes of genetic diseases, technically applicable to exome data from patients with any clinical phenotype. In brief, Haystack aggregates information from multiple databases in an R Shiny application and allows for filtering based on the aggregated data, thus minimizing the manual effort required for candidate gene selection. All source code is publicly available at GitHub^2^. We used Haystack to filter variants from our SCO patients for quality (read depth ≥ 10, variant depth ≥ 4 and frequency ≥ 40%). The remaining 2870 genes were filtered again based on genotype and allele frequency in gnomAD (MAF ≤ 0.01 for homozygous variants and MAF ≤ 0.001 for heterozygous and X-linked variants) as well as on expression levels in the testes [including only genes whose expression in testis was above 50% of its maximum expression across all tissues according to the GTEx portal (v7^3^)]. Last, genes with LoF variants present in individuals with intact spermatogenesis were excluded from the selected 949 genes, leaving a total of 721 genes with LoF variants specifically found in SCO patients. *TRIM71* was found among these genes in a patient with an SCO phenotype (subject M364). This individual’s DNA was subjected to Sanger sequencing according to standard procedures in order to confirm his *TRIM71* frameshift variant. PCR was performed using the primers: F: 5′-TGCAGGCCTAATCGATGCAT-3′; R: 5′-GAAGAAGCTGCGTTGCCCTC-3′.

For the statistical analysis of exome data, the number of alleles affected and unaffected by LoF variants in *TRIM71* in all gnomAD subjects was compared to affected and unaffected alleles in the 908 patients with quantitative spermatogenic impairment (MERGE cohort) and the 247 SCO patients (SCO subcohort). A two-sided Fisher’s exact test was performed using the Woolf logit method.

In a subsequent step, exome data of the entire MERGE cohort and the Dutch father cohort was screened for rare (see MAF cutoffs above) missense variants in *TRIM71* (NM_001039111). The Z score for *TRIM71* was obtained from gnomAD database. Predicted pathogenicity was assessed using the scoring algorithms CADD (v1^4^), PolyPhen (v2^5^), SIFT (v2^6^), and Mutation Taster (v6^7^). All variants identified by our study have been submitted to ClinVar database (accession ID SUB9045938). Known genetic causes of azoospermia, such as chromosomal aberrations and AZF deletions were excluded in all infertile patients with LoF or missense variants in *TRIM71*. Furthermore, all patients carrying LoF or missense variants in *TRIM71* (listed in Table 1) were evaluated for relevant variants in other genes (*n* = 181, Supplementary Table 2) that could explain the patients’ infertility. The majority of examined genes (*n* = 170, Supplementary Table 2A) were taken from a review on the genetics of male infertility (Oud et al., 2019). The included genes were selected based on associated phenotypes and had been rated with limited to definitive clinical evidence. The list was amended by adding 11 recently published genes (Supplementary Table 2B). Specifically, the patients’ exome data was screened for stop-gain, frameshift, and splice acceptor/donor variants as well as missense variants with a CADD score ≥ 20. Only rare variants (gnomAD MAF ≤ 0.01 for homozygous and MAF ≤ 0.001 for heterozygous variants) were taken into account. For recessive genes, variants were only considered if found in homozygous or likely compound-heterozygous state (two variants in the same gene in the same patient).

### Luciferase Reporter Assays

HEK293T cells were co-transfected with the suitable psiCHECK2 dual luciferase plasmid (Promega) and the specified Flag-tagged TRIM71 variant construct in a 1:4 ratio. The psiCHECK2 plasmids contained a specific 3′UTR sequence downstream of the Renilla Luciferase ORF (either the *CDKN1A* 3′UTR Torres-Fernández et al., 2019 or an artificial 3′UTR with 8x Let-7 binding sites Torres-Fernández et al., 2021), while the Firefly Luciferase encoded in the same plasmid was used as a normalization control. Transfected cells were harvested 48 h post-transfection and Renilla and Firefly signals were measured consecutively with the use of a luminometer (MicroLumat Plus LB96V, Berthold) and the Dual Luciferase Reporter Assay Kit (Promega) according to the manufacturer’s instructions.

## Data Availability Statement

The data analyzed in this study is subject to the following licenses/restrictions: The exome sequencing data is part of the Reproductive Genomics (MERGE) study conducted by the Institute of Reproductive Genetics, University of Münster, and contains data mostly from patients attending the Centre of Reproductive Medicine and Andrology (CeRA), University Hospital Münster. Requests to access these datasets should be directed to FT, frank.tuettelmann@ukmuenster.de.

## Ethics Statement

The studies involving human participants were reviewed and approved by the Ethics Committee of the State Medical Board and the Medical Faculty of the University of Münster. The subjects provided their written informed consent to participate in this study. Written informed consent was obtained from the individual(s) for the publication of any potentially identifiable images or data included in this article. The animal study was reviewed and approved by Landesamt f r Natur-, Umwelt- und Verbraucherschutz NRW. Patients attended the Centre of Reproductive Medicine and Andrology (CeRA) at the University Hospital M nster or the Clinic for Urology, Pediatric Urology and Andrology in Gießen. All individuals gave written informed consent and the study protocol has been approved by the appropriate ethics committees according to the Declaration of Helsinki (Münster: Kennzeichen 2010-578-f-S, Gießen: No. 26/11).

## Author Contributions

LT-F, JE, YP, and SM designed and performed experiments. LT-F and JE wrote the manuscript. SM and SS developed the germline-specific *Trim71* KO mouse. YP, SM, SS, and LT-F performed *in vivo* and *in vitro* experiments. MH and YP performed NGS Illumina MiSeq. NN and SD provided testis single-cell RNAseq data. SK and DF provided samples and histological evaluation for patients from Münster and Gießen, respectively. JE and MW developed the variant filtering tool Haystack. JE performed bioinformatics analyses. MO provided genetic data for the cohort of Dutch fathers. WK, FT, HS, and CF supervised experimental design, data analysis and manuscript writing. All authors contributed to the article and approved the submitted version.

## Conflict of Interest

LT-F held a stipend which was donated by Bayer AG. The remaining authors declare that the research was conducted in the absence of any commercial or financial relationships that could be construed as a potential conflict of interest.

## Footnotes

1 https://gnomad.broadinstitute.org

2 https://github.com/MarWoes/haystack

3 https://gtexportal.org

4 https://cadd.gs.washington.edu/

5 http://genetics.bwh.harvard.edu/pph2

6 https://sift.bii.a-star.edu.sg

7 http://www.mutationtaster.org
