# Supplementary material for: TRIM71 Deficiency Causes Germ Cell Loss During Mouse Embryogenesis and Is Associated With Human Male Infertility
Source: Front Cell Dev Biol. 2021 May 13;9:658966. doi: 10.3389/fcell.2021.658966 (PMC8155544; doi:10.3389/fcell.2021.658966)
Supplement: Supplementary file 1 [file Data_Sheet_1.pdf]

Supplementary Data

TRIM71 **deficiency causes germ cell loss during mouse embryogenesis and is associated with human male infertility**

Lucia A. Torres-Fernández*, Jana Emich*, Yasmine Port*, Sibylle Mitschka, Marius Wöste, Simon Schneider, Daniela Fietz, Manon S. Oud, Sara Di Persio, Nina Neuhaus, Sabine Kliesch, Michael Hölzel, Hubert Schorle, Corinna Friedrich, Frank Tüttelmann and Waldemar Kolanus

Submitted to Frontiers in Cell and Developmental Biology on 26.01.2021. Accepted on 30.03.2021.

Supplementary Figure 1

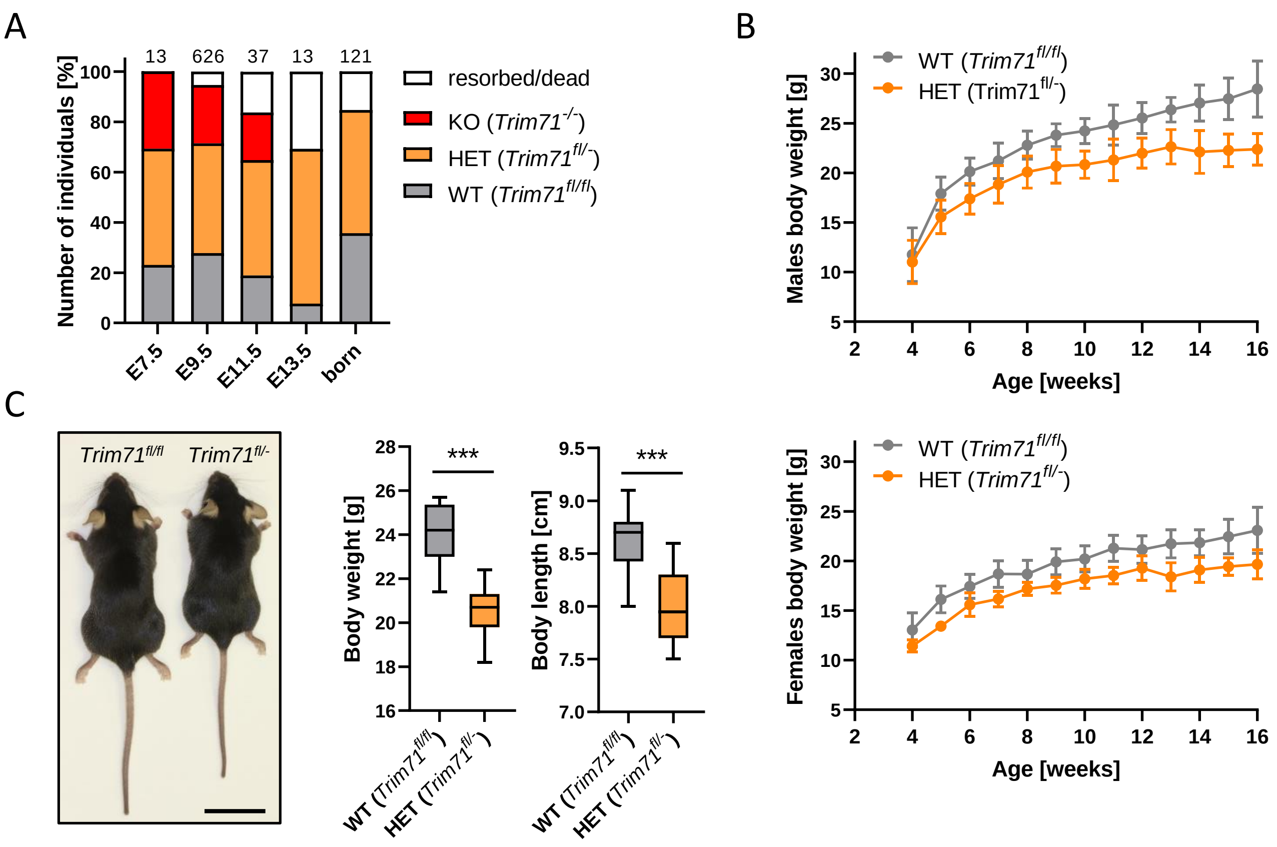

Supplementary Figure 1 (relative to Figure 1). *Trim71* heterozygous animals reveal a haploinsufficiency of *Trim71* gene. A) Genotyping statistics of embryos from *Trim71* heterozygous intercrosses depicted as percentages. Embryonic lethality of full *Trim71* knockout (KO, *Trim71*$^{-/-}$) mice is shown by an alteration of Mendelian ratios in the neonatal (born, n=121) population (***P-value < 0.005, Chi square test), with embryos resorbed between E9.5-13.5. The numbers of analyzed individuals in each case are indicated above the columns. **B)** Growth curves (weight gain over time) for *Trim71* wild type (WT, *Trim71*$^{fl/fl}$) versus *Trim71* heterozygous (HET, *Trim71*$^{fl/-}$) mice for male (top) and female (bottom) mice. Error bars represent SEM (n=3-15). Weight gain overtime was significantly different between wild type and heterozygous mice for both males and females: ***P-value < 0.005 (unpaired Student's t-test on area under curve (AUC) measurements). **C)** Representative image of adult male wild type and *Trim71* heterozygous mice accompanied by a quantification of body weight [g] and body length [cm] of adult (10-week-old) males. Scale bar represents 2 cm. Graphs represent Tukey plots (n=11-15). ***P-value < 0.005 (unpaired Student's t-test).

Supplementary Figure 2

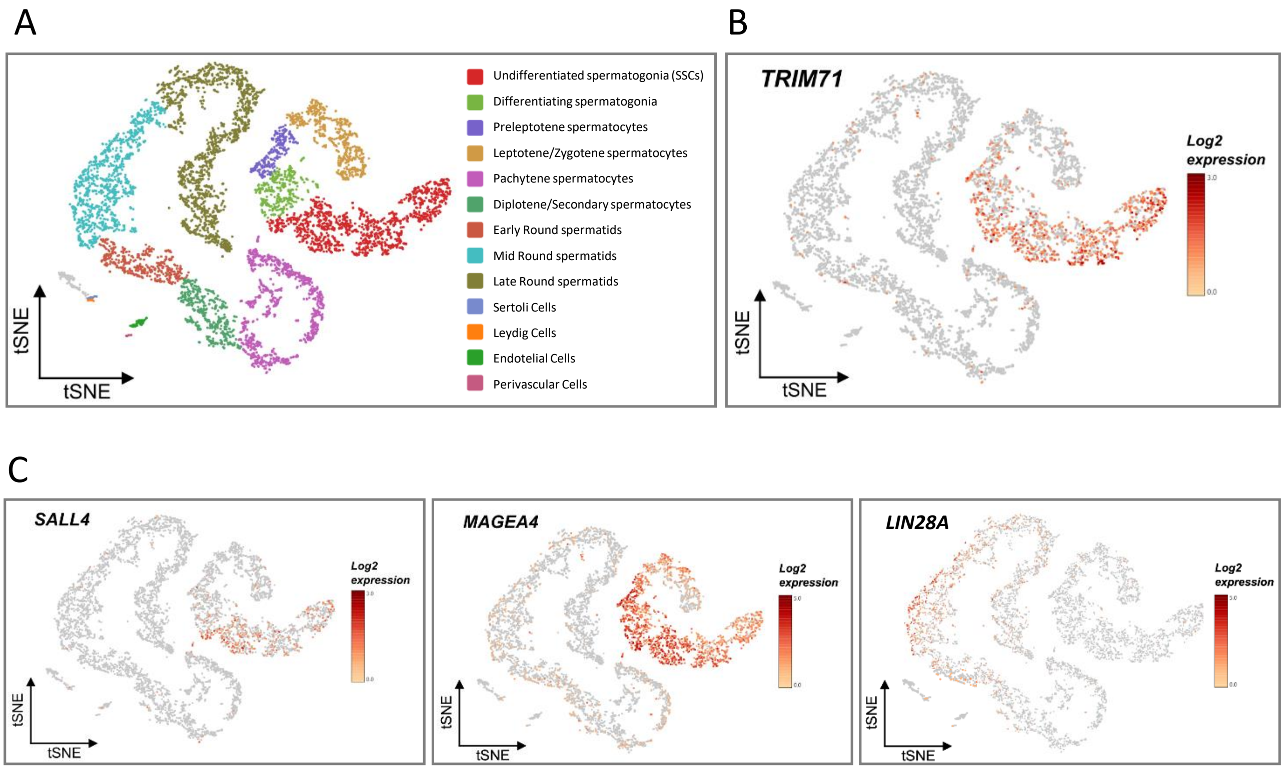

Supplementary Figure 2 (relative to Figure 1). *TRIM71* is expressed in spermatogonial stem cells (SSCs) in adult human testes. A) tSNE plot (t-distributed stochastic neighbor embedding) of single-cell transcriptome data (scRNA-seq) from human testes as published by Hermann *et al.,* 2018. Each dot represents a single cell and is colored according to its cluster identity as indicated in the figure key. **B)** Expression pattern of *TRIM71* and **C)** *SALL4*, *MEGEA4* and *LIN28A* projected on the tSNE plot of the human scRNA-seq dataset. Note that *LIN28A* is not an SSC marker in humans. Red indicates high expression and gray indicates low or no expression, as shown by the figure key.

Supplementary Figure 3

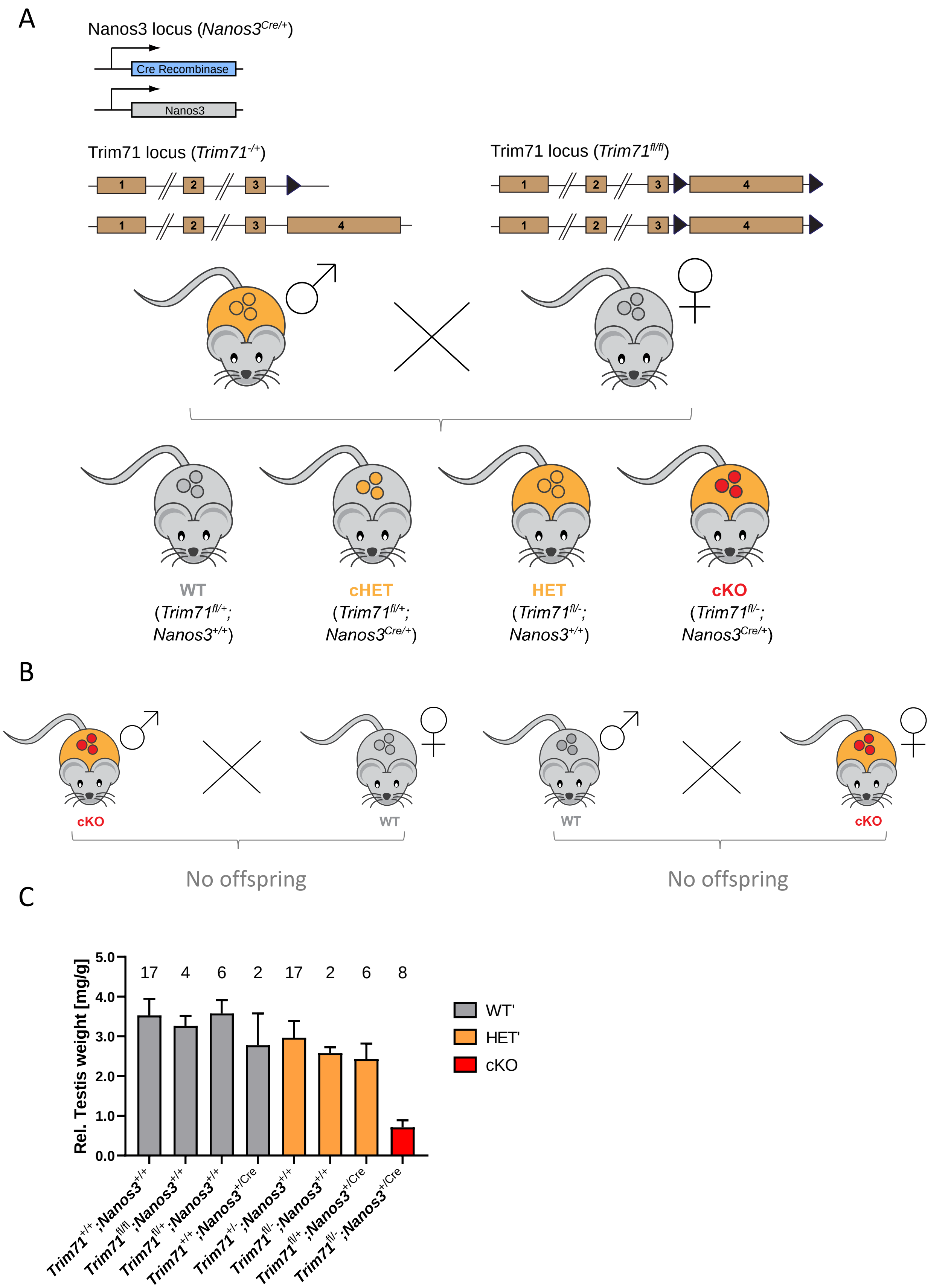

Supplementary Figure 3 (relative to Figures 2 and 3). *Trim71* is essential for fertility. A) Schematic representation for the generation of a germline-specific *Trim71* conditional knockout (cKO) mouse. Heterozygous (*Trim71*$^{+/-}$) males additionally carrying the *Nanos3*-Cre allele (*Nanos3*$^{Cre/+}$) were crossed with homozygously floxed *Trim71* (*Trim71*$^{fl/fl}$) females. The circles depicted inside the mice bodies represent the germline in adult males and females. Body and germline colors are indicative of the genotype: grey = WT, wild type (+/+, fl/fl or +/fl); orange = HET, heterozygous (+/- or fl/-) *Trim71* deletion; red = cKO, homozygous (-/-) *Trim71* deletion. **B)** Schematic representation of intercrosses for the functional characterization of *Trim71* cKO mice. No male or female germline-specific *Trim71* cKO mouse was able to generate offspring in the course of several months. **C)** Testis weight [mg] relative to total body weight [g] of adult male mice with different *Trim71* and *Nanos3* genotypes. In this case, colors are indicative of the *Trim71* genotype in the germline, regardless of *Nanos3* genotype. WT´ = wild type *Trim71* locus (+/+, fl/fl or +/fl) with different *Nanos3* genotypes; HET' = heterozygous (+/- or fl/-) *Trim71* locus with different *Nanos3* genotypes. These results are summarized in main Fig. 2C joining several *Nanos3* genotypes per *Trim71* genotype. Error bars represent SEM and the numbers of analyzed individuals in each case are indicated above the columns.

Supplementary Figure 4

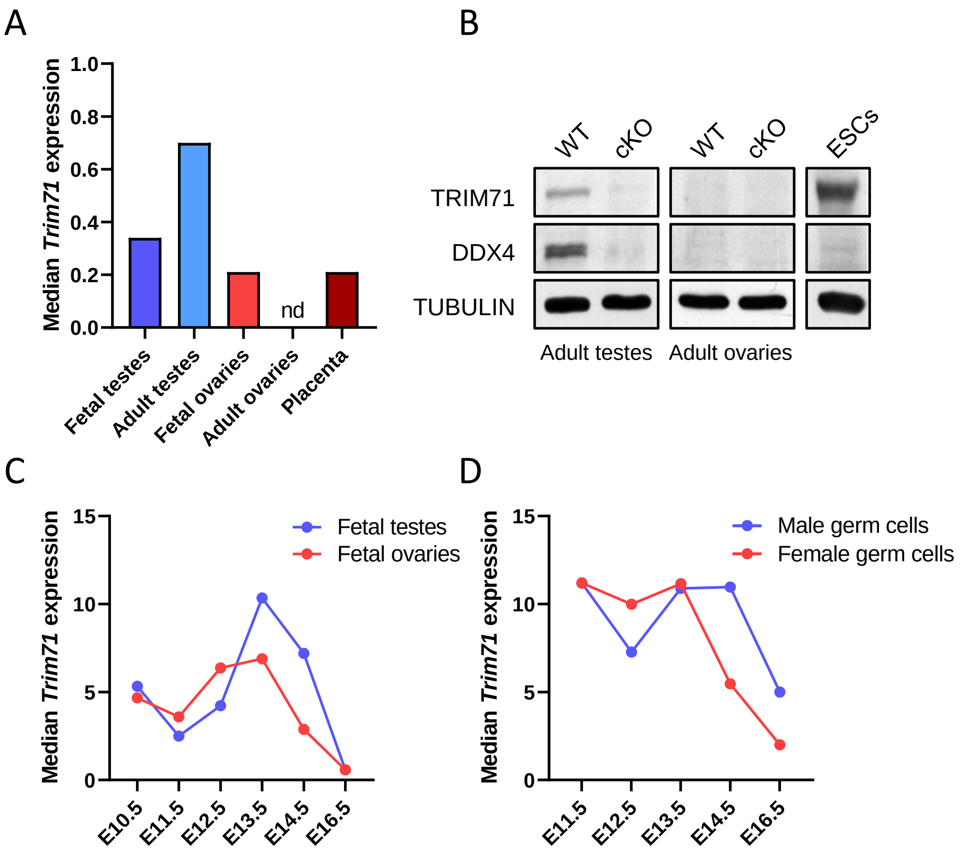

Supplementary Figure 4 (relative to Figures 2 and 3). Analysis of *Trim71* expression in available datasets reveals a common expression pattern in the embryonic germline of male and female mice. A) Median *Trim71* expression detected in fetal and adult mouse gonads as well as female placenta via Microwell-Seq as published by Han *et al.,* 2018, *Cell*. Nd = non-detected. **B)** Representative immunoblot showing TRIM71 protein expression in adult testes and ovaries from 9-month old wild type (*Trim71*$^{+/+}$; *Nanos3*$^{Cre/+}$) and *Trim71* cKO (*Trim71*$^{fl/}$; *Nanos3*$^{Cre/+}$) mice, compared to its expression in murine ESCs. DDX4 was used as a germ cell-specific marker known to be present in adult testes but not in adult ovaries (Zhang *et al.,* 2015, *Nat. Med*.). TUBULIN was used as a loading control. **C)** Median *Trim71* expression detected in murine fetal male and female gonads at different developmental stages via RNA-Seq as published by Soh *et al.,* 2015, *PLoS Genet*. and Zhao *et al.,* 2018, *Mol. Cell. Endocrinol.* **D)** Median *Trim71* expression detected in murine male and female germ cells at different developmental stages via RNA-Seq as published by Seisenberger *et al.,* 2012, *Mol. Cell.* The data depicted in A, C and D was obtained from ReproGenomics Viewer (https://rgv.genouest.org/studies/).

Supplementary Figure 5

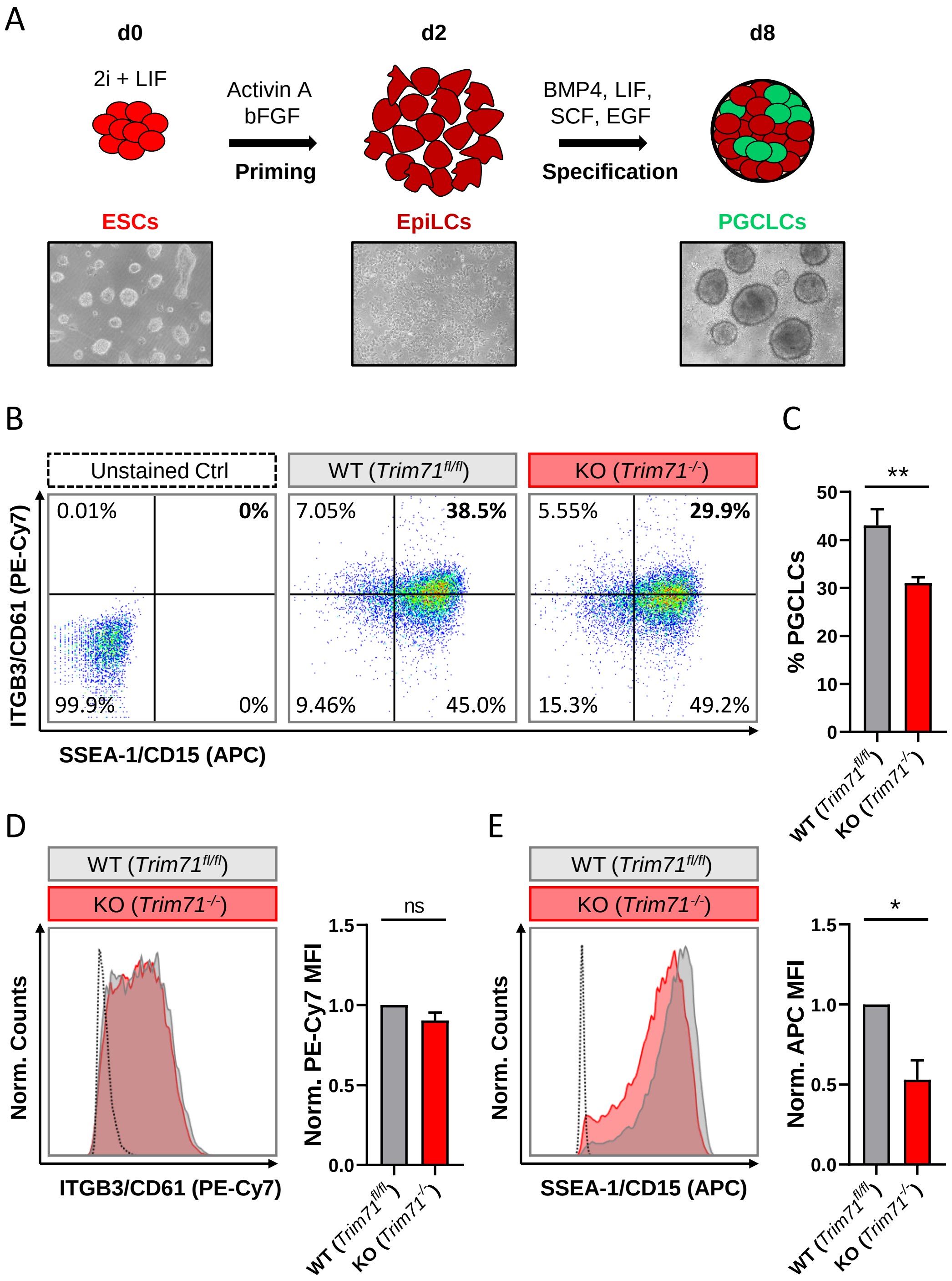

Supplementary Figure 5. *In vitro* PGC specification reveals reduced numbers of PGCLCs derived from *Trim71*-deficient ESCs. A) Schematic representation of the methodology for the *in vitro* differentiation of murine ESCs into PGC-like cells (PGCLCs) accompanied by representative microscopic images (10x magnifications) of cells at different stages of the differentiation process (d0, d2 and d8). **B)** Representative flow cytometry scatter plots from d8 cells (whole aggregates) derived from wild type (WT, *Trim71*$^{fl/fl}$) and *Trim71* knockout (KO, *Trim71*$^{-/-}$) mouse ESCs stained for the PGC-specific surface markers ITGB3/CD61 (PE-Cy7) and SSEA-1/CD15 (APC). Double positively stained cells are considered PGCLCs. **C)** Quantification of PGCLCs in several experimental replicates of B (n = 6). **P-value < 0.01 (unpaired Student's t-test). **D)** Representative histograms for individual ITGB3/CD61 (PE-Cy7) and **E)** SSEA-1/CD15 (APC) stainings of d8 cells derived from wild type (WT, *Trim71*$^{fl/fl}$) and *Trim71* knockout (KO, *Trim71*$^{-/-}$) murine ESCs accompanied by the median fluorescence intensity (MFI) quantification for the respective staining in replicate experiments (n = 6). *P-value < 0.05; ns = non-significant (paired Student's t-test). For all graphs, error bars represent the SEM. See also Suppl. Fig. 5 and 6.

Supplementary Figure 6

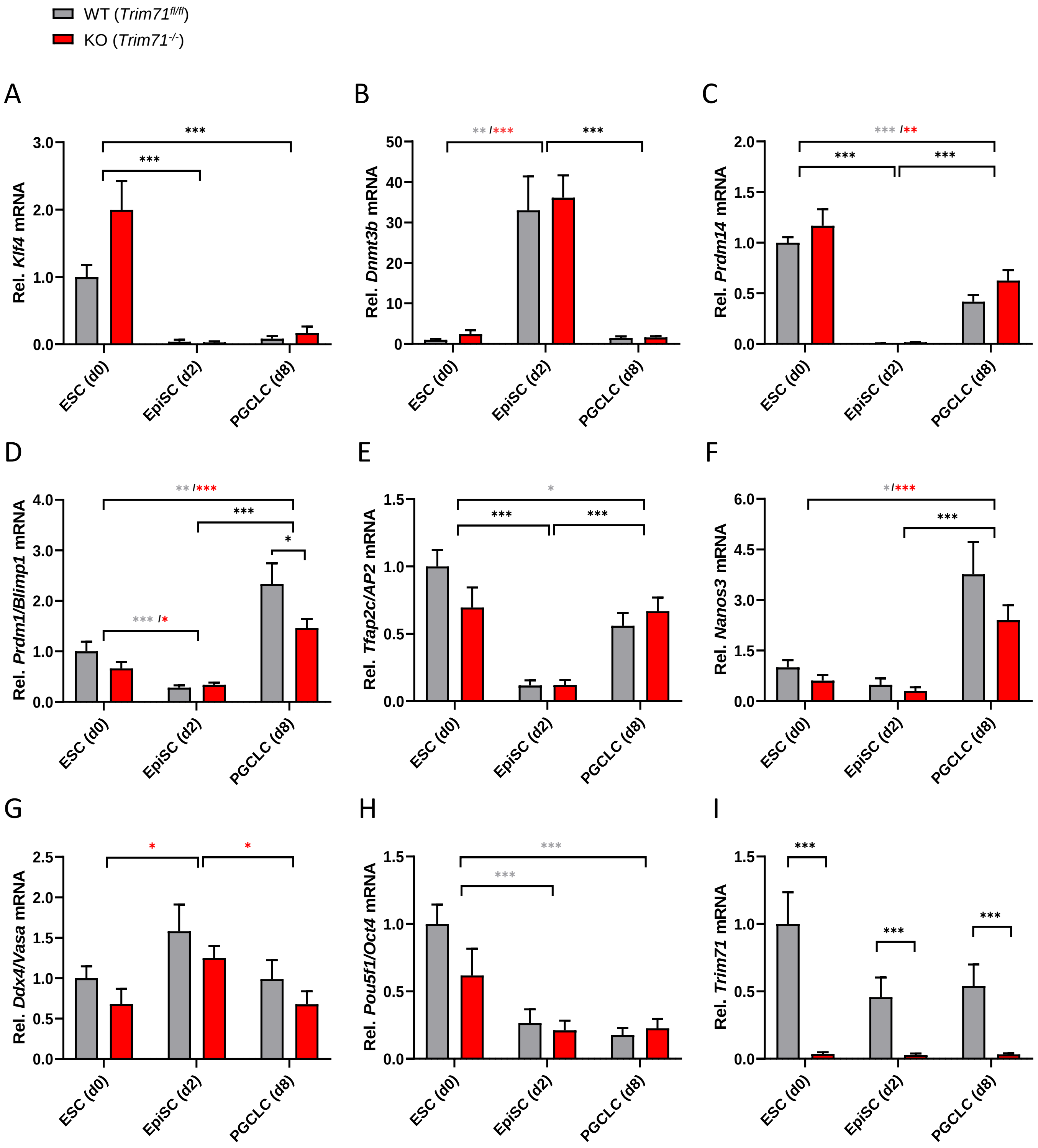

Supplementary Figure 6 (relative to Supplementary Figure 5). Monitoring *in vitro* differentiation of ESCs into PGCLCs. qRT-PCR analysis of the indicated markers to monitor the priming and specification processes of mouse ESCs into PGCLCs, measured at d0 (ESCs, naïve pluripotency), d2 (EpiLCs, primed pluripotency) and d8 (PGCLCs, representing E9.5 PGCs). As detailed in the main text, *Klf4* is a marker of naïve pluripotency and *Dnmt3b* of primed pluripotency, while *Trim71* and *Pou5f1/Oct4* expression is expected in both naïve and primed stem cells. *Prdm14, Prdm1/Blimp1, Tfap2c* and *Nanos3* are early PGC markers expected to be upregulated at d8 compared to d2. *Ddx4/Vasa* is a late PGC marker expected to be upregulated after PGC migration to the genital ridges (E10.5). For all graphs, error bars represent the SEM. ***P-value < 0.005, **P-value < 0.01; *P-value < 0.05; black stars represent equal statistical significance for both genotypes, while grey and red stars represent specific significance for wild type (WT, $Trim71^{fl/fl}$) and *Trim71* knockout (KO, $Trim71^{-/-}$) cells, respectively (unpaired Student's t-test).

Supplementary Figure 7

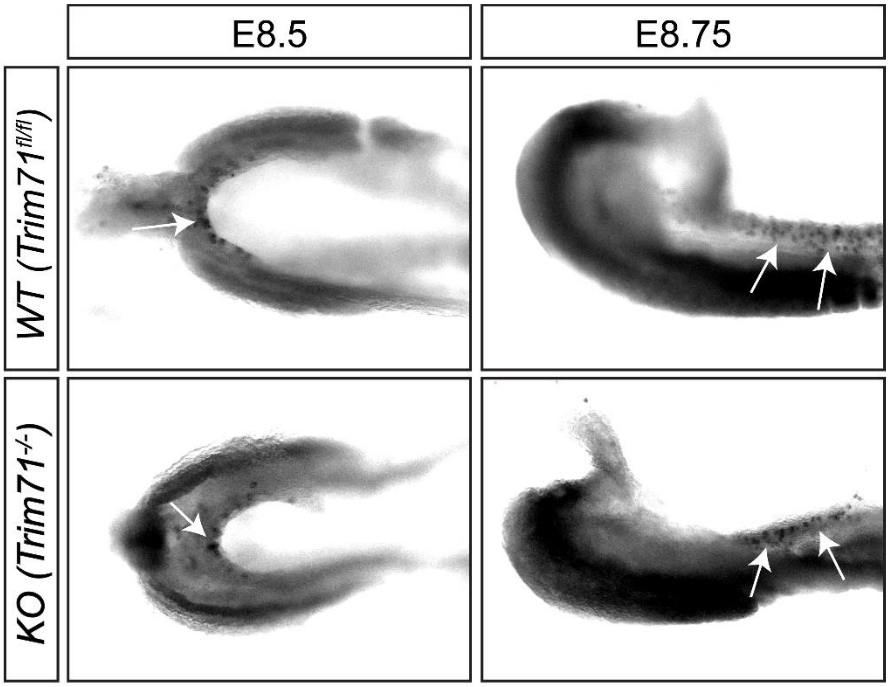

Supplementary Figure 7. *Trim71* deficiency does not impair *in vivo* PGC migration towards the genital ridges. Alkaline phosphatase (AP) staining in wild type (WT, *Trim71*$^{fl/fl}$) and full *Trim71* knockout (KO, *Trim71*$^{-/-}$) embryos at stages E8.5-8.75 (n = 4-6 embryos per genotype, from two different mothers). PGCs (AP-positive cells) are marked with white arrows in posterior hindgut pieces from E8.5 (dorsal view) and E8.75 (lateral view) sibling embryos from the mating of full heterozygous animals (HET, *Trim71*$^{fl/-}$).

Supplementary Figure 8

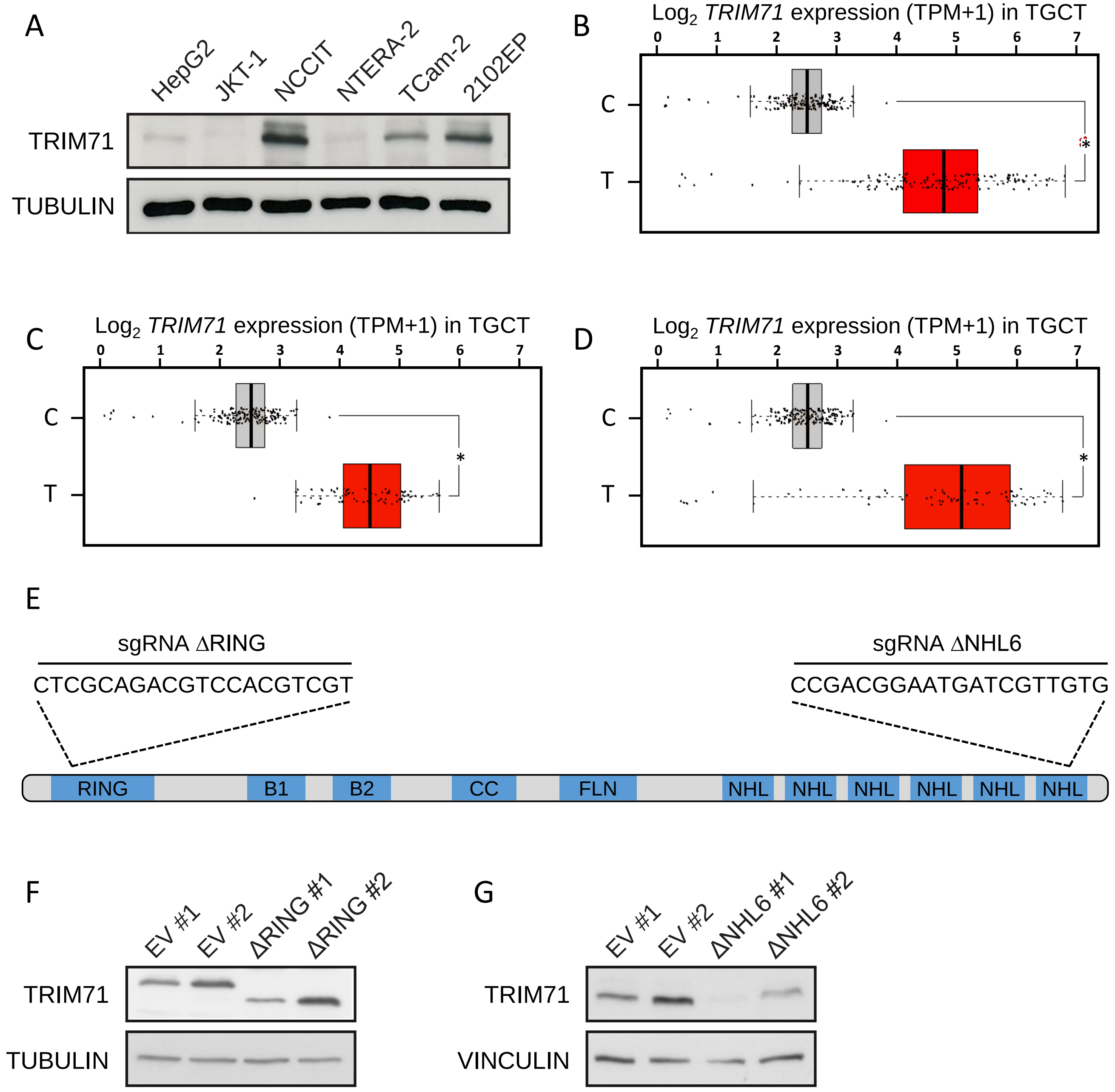

Supplementary Figure 8 (relative to Figure 5). Generation of *TRIM71* mutations via CRISPR/Cas9 in NCCIT cells. A) Representative immunoblot showing TRIM71 protein expression in several germ cell tumor (GCT)-derived embryonic carcinoma cell lines. TUBULIN was used as a loading control. **B)** *TRIM71* expression is upregulated in human testicular germ cell tumor (TGCT) patients. Box plot graph was obtained from GEPIA (http://gepia2.cancer-pku.cn) and shows *TRIM71* mRNA levels in human control (C, n = 165) and tumor (T, n = 196) samples. Tumor samples include seminoma (n = 131) and non-seminoma (n = 65) TGCT subtypes. TPM = transcript counts per million. **C)** *TRIM71* expression in TGCT patients from B, depicting control (C, n=165) *versus* only seminoma tumor (T, n = 131) samples. **D)** *TRIM71* expression in TGCT patients from B, depicting control (C, n = 165) *versus* only non-seminoma tumor (T, n = 65) samples. **E)** Schematic representation of TRIM71 structural domain organization indicating the location and target sequences (single guide RNA = sgRNA) for the generation of NCCIT cells with *TRIM71* frameshift mutations in the RING domain (ΔRING) and the NHL domain (ΔNHL6) via CRISPR/Cas9. **F)** Representative immunoblot showing TRIM71 protein in two single NCCIT clones targeted with sgRNA ΔRING including wild type cells transfected with an empty vector (EV). Of note, *TRIM71* RING mutant NCCIT cells (ΔRING) express a RINGless protein of 83 kDa due to the usage of an alternative in-frame ATG codon located downstream of the targeted sequence. **G)** Representative immunoblot showing TRIM71 protein in two single NCCIT clones targeted with sgRNA ΔNHL6 including wild type cells transfected with an empty vector (EV). For *TRIM71* NHL mutant NCCIT cells (ΔNHL6) the deletion of the last C-terminal 24 aa (clone #1) renders an unstable protein which seems to be degraded while the deletion of the last C-terminal 18 aa (clone #2) renders a stable C-terminal truncated protein of 92 kDa.

Supplementary Figure 9

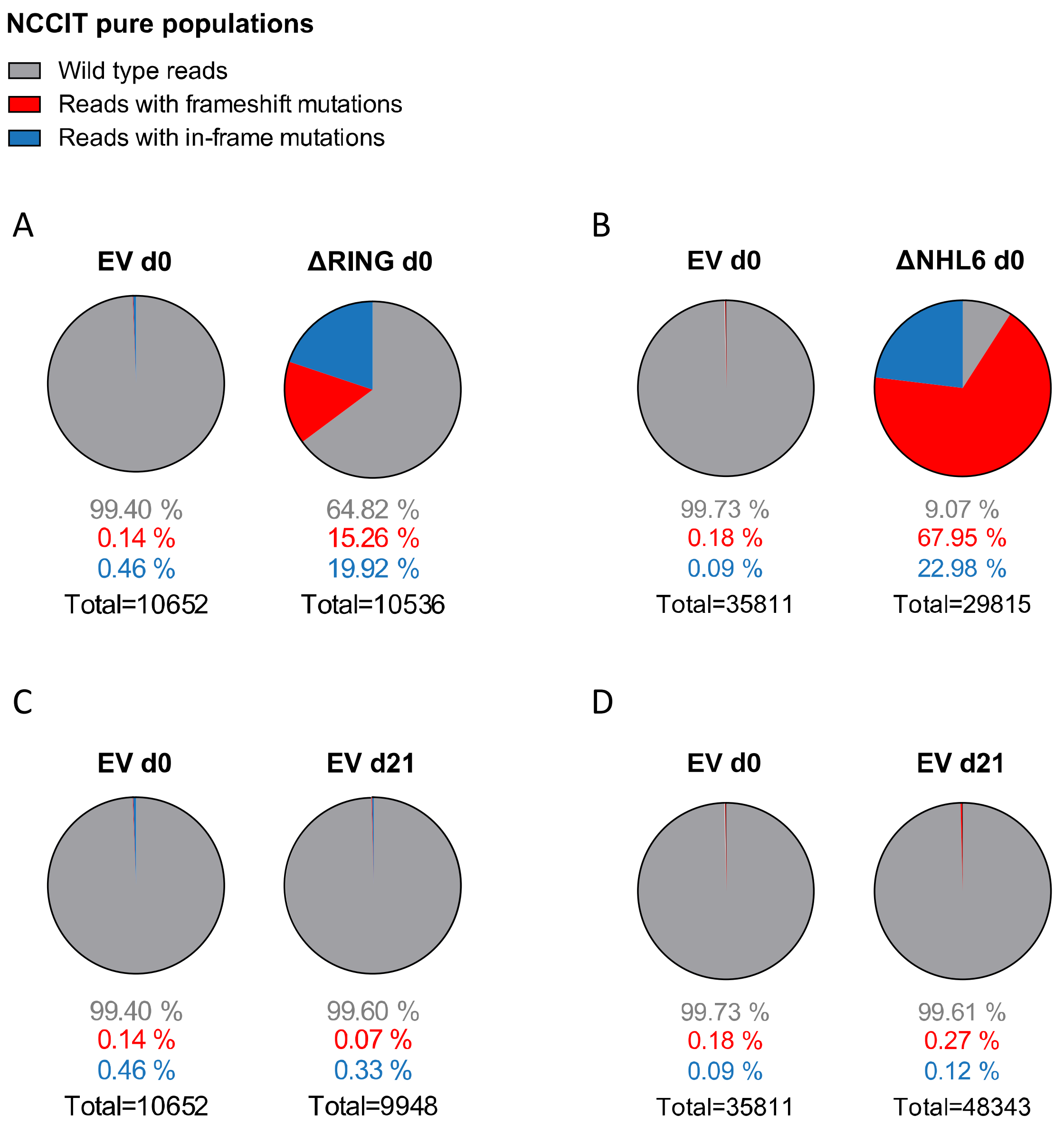

Supplementary Figure 9 (relative to Figure 5). NGS analysis via Illumina MiSeq platform of pure NCCIT wild type and *TRIM71* mutant populations. A) Pie charts showing allele frequencies at d0 for wild type NCCIT cells transfected with an empty vector (EV) and mutant NCCIT cells transfected with a sgRNA targeting TRIM71's RING domain (ΔRING). The depicted pure populations were then mixed 1:1 and cultured for growth competition assays in which allele frequencies were measured at different time points via Illumina Miseq analysis (corresponding to Fig. 6A). **B)** Pie charts showing allele frequencies at d0 for wild type NCCIT cells transfected with an empty vector (EV) and mutant NCCIT cells transfected with a sgRNA targeting TRIM71's NHL domain (ΔNHL6). The depicted pure populations were then mixed 1:1 and cultured for growth competition assays in which allele frequencies were measured at different time points via Illumina Miseq analysis (corresponding to Fig. 6B). **C)** Pie charts showing allele frequencies at d0 and d21 for wild type NCCIT cells transfected with an empty vector (EV) from A. **E)** Pie charts showing allele frequencies at d0 and d21 for NCCIT cells transfected with an empty vector (EV) from B.

Supplementary Figure 10

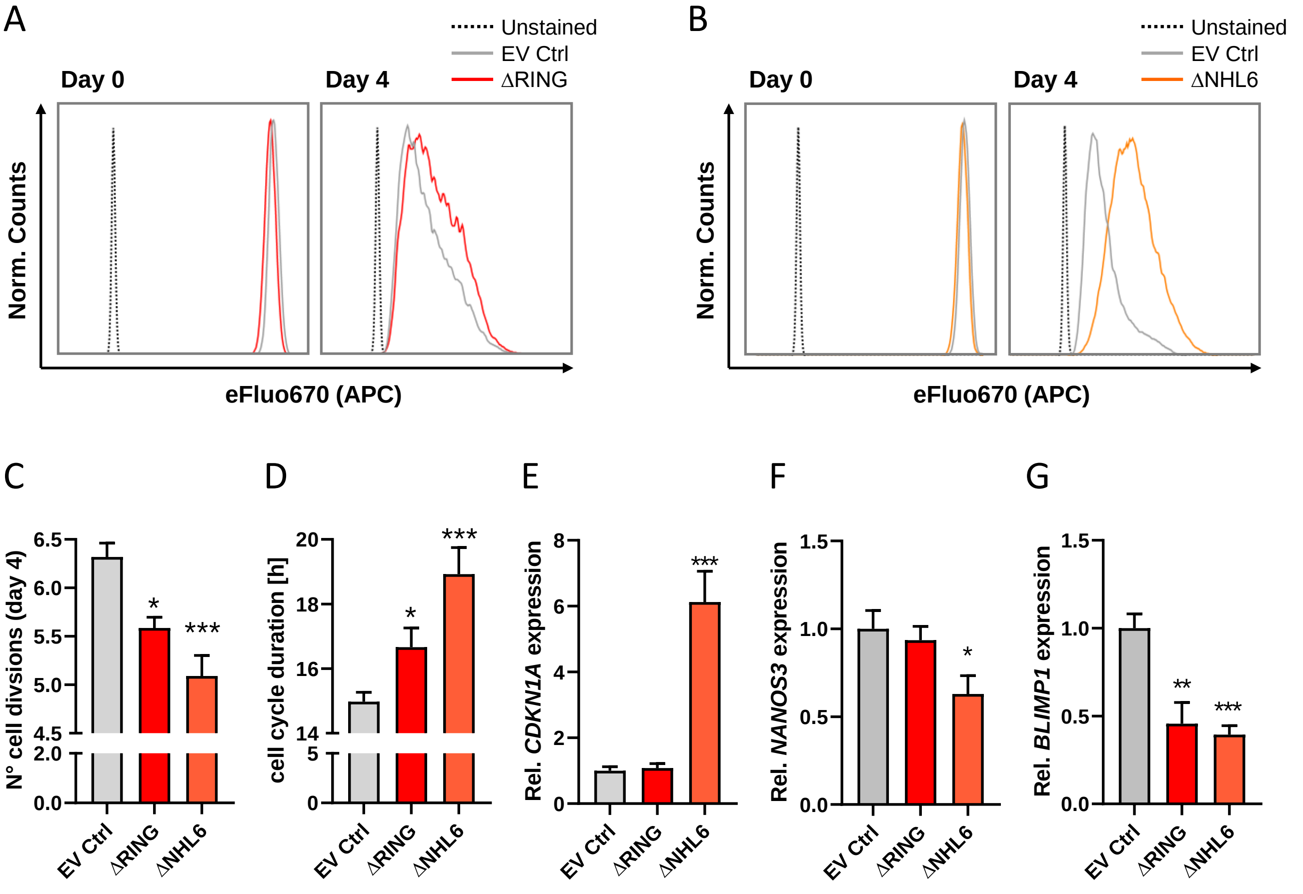

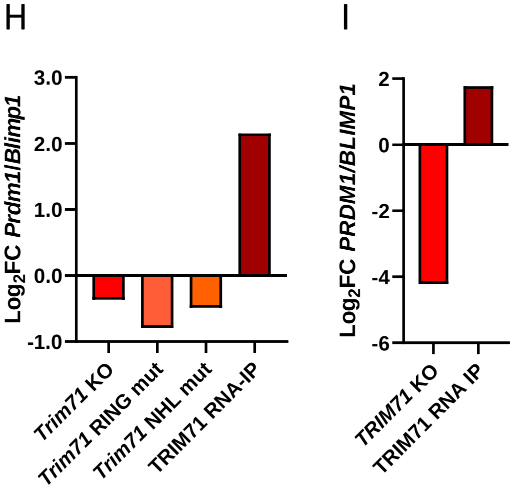

J

Gene Name	Position (bp)	Loop sequence	Strength
Prdm1/Blimp1	421	UACCA	Minimal-Weak
Prdm1/Blimp1	461	CAAAG	Minimal-Weak
Prdm1/Blimp1	1103	UUUGA	Minimal-Weak
Prdm1/Blimp1	1628	CAAAG	Minimal-Weak
Prdm1/Blimp1	1759	UACAA	Minimal-Weak
Prdm1/Blimp1	2880	UAUAA	Medium-Strong
Prdm1/Blimp1	2991	CCUAG	Minimal-Weak
Prdm1/Blimp1	3050	UCAAA	Minimal-Weak
Prdm1/Blimp1	3339	CUCAG	Minimal-Weak
Prdm1/Blimp1	3482	UCUGA	Medium-Strong

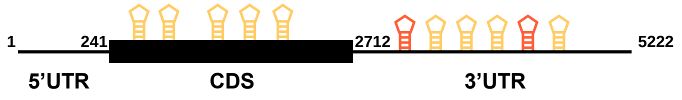

Supplementary Figure 10 (relative to Figure 5). ***TRIM71*** **mutant NCCIT cells show proliferation defects and deregulation of PGC markers. A)** Representative flow cytometry eFluo670 (APC) histograms for the proliferation assays with wild type (EV Ctrl) and TRIM71 mutant ΔRING or **B)** ΔNHL6 NCCIT cells, showing comparable fluorescence intensity for the different cell populations after staining (day 0) and differences in fluorescence intensity at the end of the experiment (day 4). A slower loss of fluorescence intensity over time is apparent in *TRIM71* mutant NCCIT histograms as compared to wild type cells in each case, and is indicative of a decreased proliferation (n = 3-6). **C)** Number of cell divisions undergone at the end of the experiment (day 4) by wild type (EV Ctrl) and TRIM71 mutant ΔRING and ΔNHL6 NCCIT cells. The number of divisions was calculated assuming that the MFI of the proliferation dye eFluo670 (APC) decreases by half upon each cell division, as $\log_2 [(MFI_{day0} - MFI_{unstained})/(MFI_{day4} - MFI_{unstained})]$. (n = 3-6). **D)** Average duration of the cell cycle in hours (h) for wild type (EV Ctrl) and TRIM71 mutant ΔRING and ΔNHL6 NCCIT cells, calculated by dividing the total experimental time (4 days = 96 h) by the number of cell divisions (n = 3-6). **E)** RT-qPCR measurements for the expression of *CDKN1A*, **F)** *NANOS3* and **G)** *BLIMP1* in wild type (EV Ctrl) and TRIM71 mutant ΔRING and ΔNHL6 NCCIT cells (n = 3-6). For all graphs, error bars represent the SEM. ***P-value < 0.005, **P-value < 0.01; *P-value < 0.05 (unpaired Student's t-test). **H)** Fold change expression (Log2FC) of *Prdm1/Blimp1 in Trim71 KO,* RING mutant (C12LC15A) and NHL mutant (R738A) mouse ESCs (in each case relative to wild type ESCs), accompanied by the fold enrichment of *Prdm1/Blimp1* mRNA co-precipitated with full-length TRIM71 wild type protein via RNA-IPs in mouse ESCs, as published by Welte *et al.,* 2019, *Genes Dev*. The TRIM71 RING mutant C12LC15A contains point mutations in two catalytic cysteines which impair TRIM71's role as an E3 ligase, and the TRIM71 NHL mutant R738A contains a point mutation which impairs its ability to bind mRNAs. **I)** Fold change expression (Log2FC) of *PRDM1/BLIMP1 in Trim71 KO* human hepatocellular carcinoma (HCC) Huh7 cells relative to wild type Huh7 cells, as published by Welte *et al.,* 2019, *Genes Dev*., accompanied by the fold enrichment of *PRDM1/BLIMP1* mRNA co-precipitated with full-length TRIM71 wild type protein via RNA-IPs in HCC HepG2 cells, as published by Foster *et al.,* 2020, *Cell cycle*. The data in H and I supports a TRIM71-mediated positive regulation of *BLIMP1* expression via direct binding and stabilization of its mRNA. **J)** TRIM71-binding sites identified for *Blimp1* mRNA in ESCs as published by Welte *et al.,* 2019. The table (top) information was extracted and adapted from Welte *et al.,* 2019 supplementary materials and used to depict the schematic representation of TRIM71-binding sites along the *Blimp1* mRNA (bottom). The hairpin color correlates with the hairpin strength. UTR = untranslated region; CDS = coding sequence.

Supplementary Figure 11

A

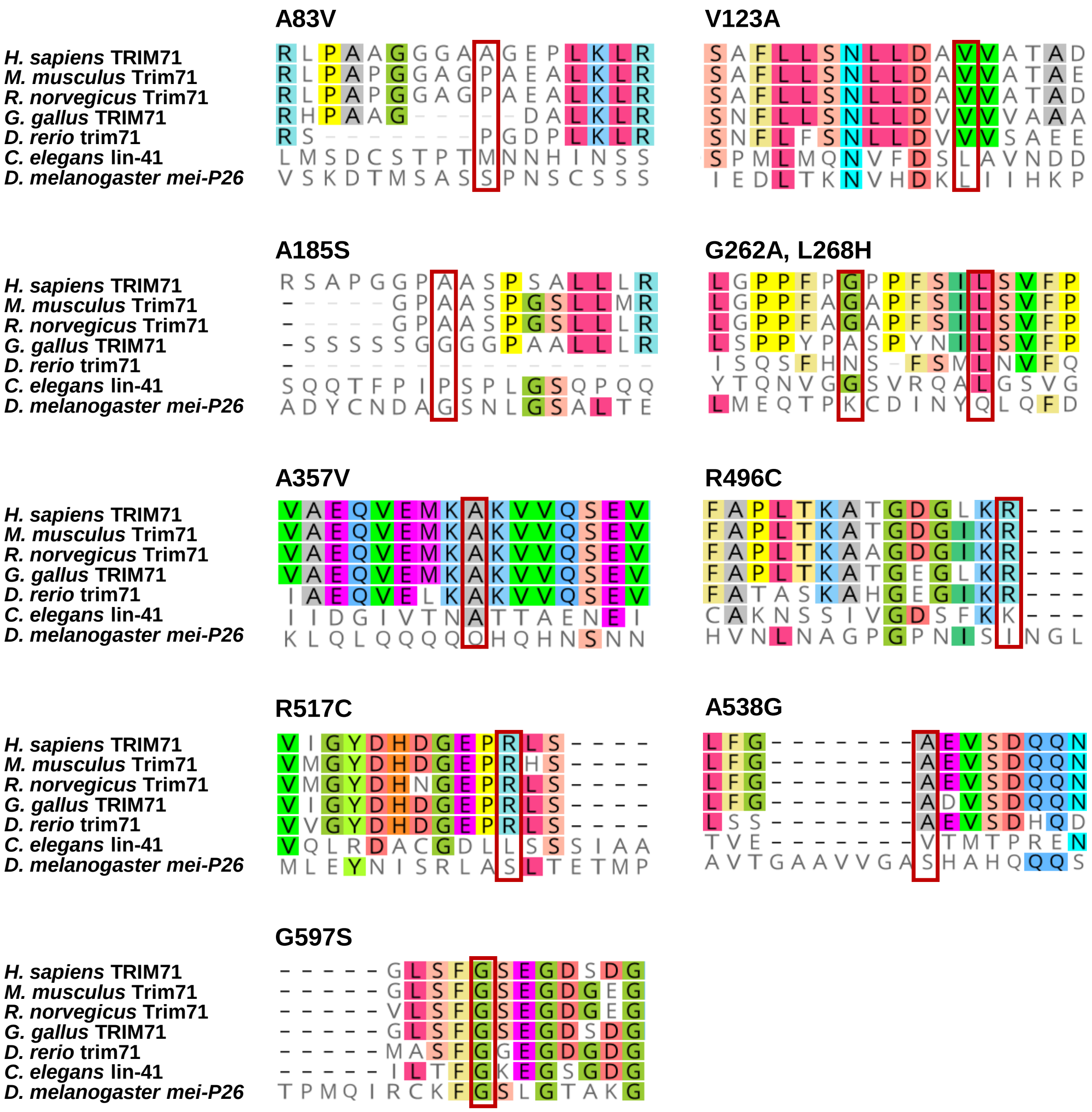

B

***TRIM71* LoF variant c224_240dup/p.(Gly81CysfsTer24)**

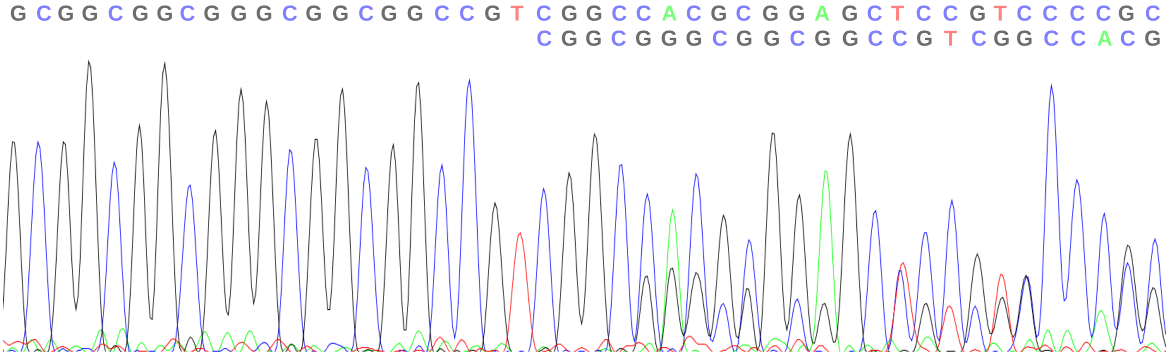

Supplementary Figure 11 (relative to Figure 6). Analysis of the evolutionary conservation of *TRIM71* genetic variants found in male infertile patients. A) Sequence conservation for the *TRIM71* genetic variants found in individuals of the MERGE cohort, obtained from MEGA/ClustalW and Geneious Prime. Affected amino acids are indicated in red boxes across all examined vertebrate and invertebrate species. **B)** Electropherogram resulting from Sanger sequencing of DNA from an SCO patient (subject M364) showing the duplication c.224_240dup resulting in the frameshift p.(Gly81CysfsTer24) in *TRIM71* locus (reverse strand).

Supplementary Figure 12

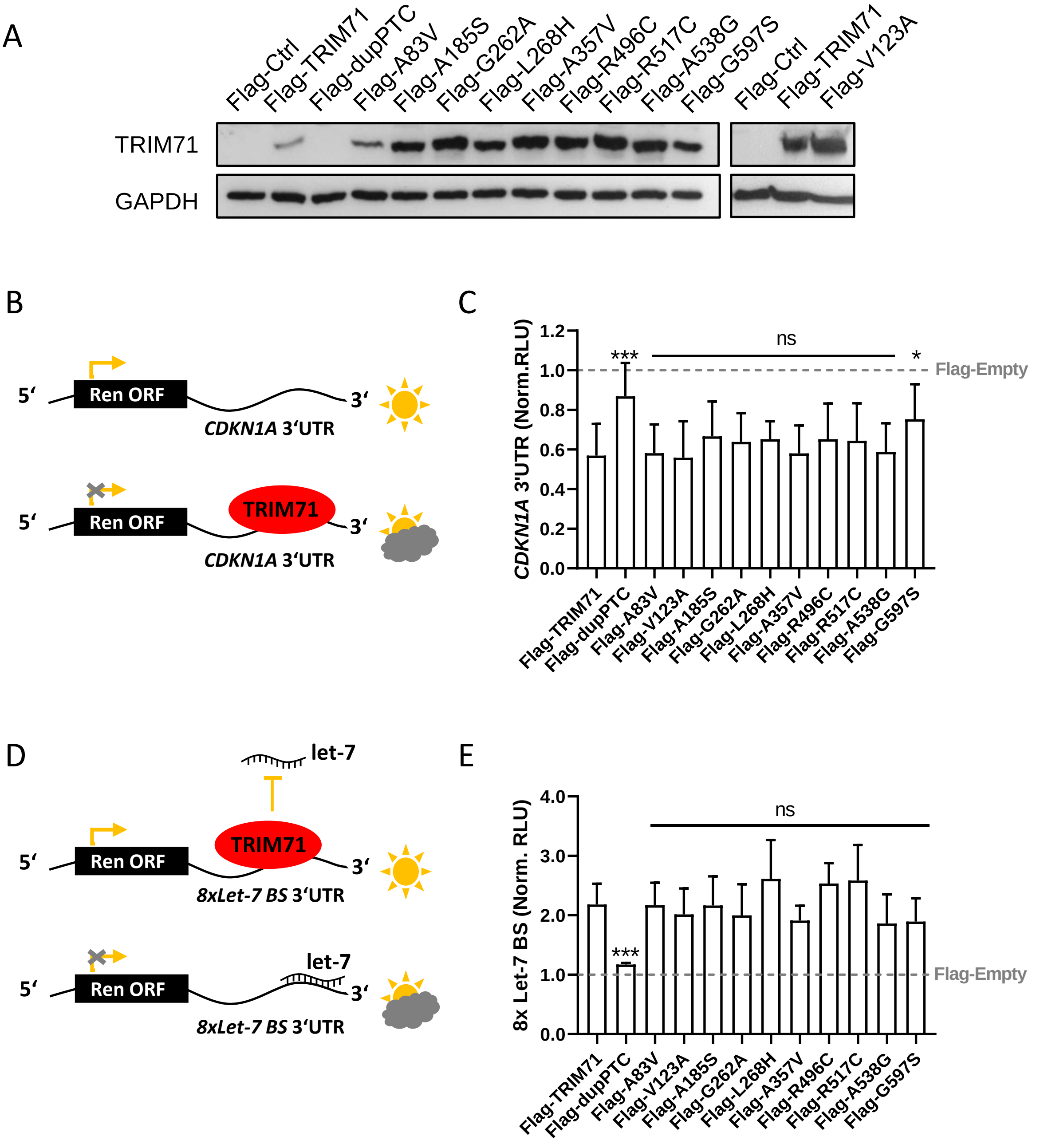

Supplementary Figure 12 (relative to Figure 6). Functional characterization of the human *TRIM71* variants identified in infertile men via exome sequencing analysis. A) Representative immunoblot analysis showing the expression of all TRIM71 variants identified in a cohort of infertile men with severely impaired spermatogenesis. All variants were cloned as Flag-tagged constructs in mammalian expression vectors and overexpressed in HEK293T cells. Protein lysates were prepared 48 hpt. GAPDH was used as loading control. **B)** Schematic representation of *CDKN1A* 3'UTR luciferase reporter assays. TRIM71 is able to repress the expression of a Renilla luciferase under the control of the human full-length *CDKN1A* 3'UTR resulting in a decrease of the reporter activity, as published by Torres-Fernández *et al.,* 2019, *Nucleic Acids Res*. **C)** Luciferase reporter assays showing the repression of the *CDKN1A* 3'UTR reporter by different TRIM71 variants compared to that of the full-length wild type TRIM71, all of which were normalized to the repression mediated by a Flag-empty control construct (depicted by the grey baseline). (n = 5-10). **D)** Schematic representation of let-7 miRNA luciferase reporter assays. TRIM71 is able to repress the activity of the miRNA let-7 via direct binding and stabilization of let-7 target mRNAs, as published by Torres-Fernández *et al.,* 2021, *BioRxiv*. TRIM71-mediated let-7 activity inhibition results in the derepression and increased activity of a Renilla luciferase under the control of an artificial 3'UTR containing 8x let-7 binding sites. **E)** Luciferase reporter assays showing the derepression of the let-7 reporter by different TRIM71 variants compared to that of the full-length wild type TRIM71, all of which were normalized to the repression mediated by a Flag-empty control construct (depicted by the grey baseline). (n = 3-10). Ren = Renilla Luciferase; Norm. RLU = Normalized Relative Light Units; ORF = Open Reading Frame. UTR = untranslated region; BS = binding sites. DupPTC = duplication generating a premature termination codon. For all graphs, error bars represent the SD. ***P-value < 0.005; *P-value < 0.05; ns = non-significant (One-way ANOVA comparing each TRIM71 variant to the full-length wild type TRIM71).

Supplementary Table 1

Variant (cDNA)	Variant (protein)	Classification	Fulfilled criteria
c.224_240dup	p.(Gly81CysfsTer24)	likely pathogenic	PS3, PM2, PP3
c.248C>T	p.(Ala83Val)	VUS	PM1, PM2, BP4
c.368T>C	p.(Val123Ala)	VUS	PM2, PP3
c.553G>T	p.(Ala185Ser)	likely benign	BS2, BP4
c.785G>C	p.(Gly262Ala)	likely benign	BS2, BP4
c.803T>A	p.(Leu268His)	VUS	PM2
c.1070C>T	p.(Ala357Val)	VUS	BP5*
c.1486C>T	p.(Arg496Cys)	VUS	PM1, PP3
c.1549C>T	p.(Arg517Cys)	VUS	PM1, PP3, BS2
c.1613C>G	p.(Ala538Gly)	VUS	PM1, PM2
c.1789G>A	p.(Gly597Ser)	VUS	PM1, PP3, BS2

Supplementary Table 1 (relative to Figure 6). Classification of identified *TRIM71* variants according to ACMG/AMP guidelines. Fulfilled classification criteria are indicated for each variant. VUS = Variant of uncertain significance. Abbreviations in accordance with ACMG: PS = pathogenic strong, PM = pathogenic moderate, PP = pathogenic supporting, BP = benign supporting, BS = benign strong. *This TRIM71 variant was found in a patient with alternative molecular cause for disease, namely a frameshift variant in *SYCP2* gene (see also Suppl. Table 2).

Supplementary Table 2

**Supplementary Table 2A. Infertility-associated genes (Reviewed in Oud *et al.,* 2019. Human Reproduction)**								
ABCA1	CCDC141	DMRT1	FGFR1	INSL3	NOS1	REC8	SPO11	UBE2B
ADGRG2	CCDC155	DNAAF2	FLNA	JAG1	NOTCH1	RELN	SRA1	UBR2
AK7	CCDC39	DNAAF4	FSHB	KDM3A	NR0B1	RNF220	SRD5A2	USP26
AKAP4	CCDC40	DNAAF5	FSHR	KISS1R	NR5A1	RSPH1	SRY	VAMP7
AMH	CDC14A	DNAH1	FSIP2	KLHL10	NRAS	RSPH3	STK36	WDR11
AMHR2	CEP135	DNAH9	GALNTL5	LHB	NSMF	RSPH9	STX2	WDR66
ANOS1	CEP290	DNAI1	GAS8	LHCGR	PANK2	RSPO1	SUN5	WT1
APOA1	CFAP43	DNAI2	GATA4	LRRC6	PDHA2	SECISBP2	SYCE1	XRCC2
AR	CFAP44	DNAJB13	GH1	MAGEB4	PIH1D3	SEMA3A	SYCP3	ZMYND15
AURKC	CFAP69	DNMT1	GNRH1	MAMLD1	PKD1	SLC26A3	TAC3	ZPBP
BMP4	CFTR	DNMT3B	GNRHR	MAP2K2	PLCZ1	SLIT2	TACR3	
BMP7	CHD7	DPY19L2	GTF2H3	MAP3K1	PLEKHA5	SOS1	TAF4B	
BNC2	CYP11A1	E2F1	HAUS7	MC4R	PLK4	SOX10	TDRD6	
BRAF	CYP11B1	ERBB4	HESX1	MEI1	PLXNA1	SOX2	TDRD7	
BRDT	CYP17A1	FANCA	HS6ST1	MEIOB	PMFBP1	SOX3	TDRD9	
BSCL2	CYP19A1	FANCM	HSD17B3	MNS1	POLR3B	SOX8	TEX11	
C11orf70	CYP21A2	FATE1	HSD3B2	MTOR	PROK2	SOX9	TEX14	
CATSPER1	DCC	FEZF1	HSF2	NANOS2	PROKR2	SPAG17	TEX15	
CATSPERE	DHH	FGF17	HYDIN	NLRP3	PSMC3IP	SPATA16	TRIM37	
CCDC103	DMC1	FGF8	IL17RD	NNT	RBMXL2	SPINK2	TTLL5	

**Supplementary Table 2B. Infertility-associated genes (reported recently in independent studies)**	
ADAD2	Krausz et al., 2020. Genetics in Medicine
M1AP	Wyrwoll et al., 2020. AJHG
MSH4	Krausz et al., 2020. Genetics in Medicine
RAD21L1	Krausz et al., 2020. Genetics in Medicine
RNF212	Riera-Escamilla et al., 2019. Human Reproduction
SHOC1	Krausz et al., 2020. Genetics in Medicine
STAG3	Riera-Escamilla et al., 2019. Human Reproduction. Van der Bijl et al., 2019. Human Reproduction
SYCP2	Schilit et al., 2020. AJHG
TERB1	Krausz et al., 2020. Genetics in Medicine & Salas-Huetos et al., 2020. Human Genetics
TERB2	Salas-Huetos et al., 2020. Human Genetics
MAJIN	Salas-Huetos et al., 2020. Human Genetics

Supplementary Table 2C. Variants identified in infertility-associated genes in patients with TRIM71 variants

Subject ID	*TRIM71* variant	Infertility-associated gene	Variant
M1686	c.1070C>T/p.(Ala357Val)	*SYCP2*	heterozygous, frameshift
M1083	c.1486C>T/p.(Arg496Cys)	*NNT**	heterozygous, missense
M468	c.1549C>T/p.(Arg517Cys)	*TERB1**	homozygous, missense
M468	c.1549C>T/p.(Arg517Cys)	NOTCH1*	heterozygous, missense
M468	c.1549C>T/p.(Arg517Cys)	TDRD7*	heterozygous, missense
M754	c.1789G>A/p.(Gly597Ser)	*HSD3B2**	heterozygous, missense
M2141	c.1789G>A(p.(Gly597Ser)	*CCDC141**	heterozygous, missense

Supplementary Table 2 (relative to Figure 6). Analysis of exome data from infertile men carrying *TRIM71* variants for the identification of variants in other genes previously associated with male infertility in humans. *Variants of uncertain significance.

Supplementary Table 3

Supplementary Table 3A. Genotyping primers	
Trim71_F1	5'-GAAAGGAGGCTAGCCAAAGG-3'
Trim71_R1	5'-ATGCTGTACGGTAGGAGTCTTCC-3'
Trim71_R2	5'-CACACAAAAAACCAACACACAG-3'
Nanos3_F1	5'-CCAGCCATGGGGACTTTC-3'
Nanos3_R1	5'-GGGACTGATAGATGGCAC-3'
Nanos3_R2	5'-CAGAGGCCACTTGTGTAGCG-3'
**Supplementary Table 3B. qRT-PCR primers (SYBR Green)**	
Klf4_F	5'-GCGAACTCACACAGGCGAGAAAC-3'
Klf4_R	5'-TCGCTTCCTCTTCCTCCGACA-3'
Prdm14_F	5'-CAGCGACTTCATTGCCAAAGGAG-3'
Prdm14_R	5'-GCCGTCGATAAAATGGCTCAGG-3'
Prdm1/Blimp1_F	5'-ACCCCTCATCGGTGAAGTCTACA-3'
Prdm1/Blimp1_R	5'-CTCCTCTCTGGAATAGATCCGCCA-3'
Ddx4/Vasa_F	5'-TCATACTTGCAGGACGAGATTTG-3'
Ddx4/Vasa_R	5'-AACGACTGGCAGTTATTCCATC-3'
18 sRNA_F	5'-GTAACCCGTTGAACCCCATTC-3'
18 sRNA_R	5'-CCATCCAATCGGTAGTAGCGAC-3'
TFAP2C_F	5'-CCCATCGAGGTCTTCTGCTC-3'
TFAP2C_R	5'-AGAGTCACATGAGCGGCTTT-3'
DDX4/VASA_F	5'-TCATACTTGCAGGACGAGATTTG-3'
DDX4/VASA_R	5'-AACGACTGGCAGTTATTCCATC-3'
**Supplementary Table 3C. qRT-PCR probes (TaqMan)**	
Trim71	Mm01341471_m1
Sall4	Mm00453037_s1
Lin28a	Mm00524077_m1
Lin28b	Mm01190673_m1
Pou5f1/Oct4	Mm03053917_g1
Dmnt3b	Mm01240113_m1
Tfap2c	Mm00493473_m1
Nanos3	Mm00808138_m1
Hprt	Mm03024075_m1
TRIM71	Hs01394933_m1
CDKN1A	Hs00355782_m1
PRDM1/BLIMP1	Hs_00153357
NANOS3	Hs_00928455
**Supplementary Table 3D. siRNAs**	
siCtrl (Renilla)	5'-AAACATGCAGAAAATGCTG-3'
siTRIM71#1	5'-CCGTGTGCGACCAGAAAGTA-3'
siTRIM71#2	5'-CCAGATCTGCTTGCTGTGCAA-5'

Supplementary Table 3 (relative to Methods). Primers and Probes used for the amplification of murine and human genes in genotyping or qRT-PCR analiysis, and siRNAs sequences used to knock down human *TRIM71* expression in TCam-2 cells. F = forward; R = reverse. TaqMan probes were acquired from Applied Biosystems (Thermo Fischer Scientific).

Supplementary Table 4

Supplementary Table 4A. Primary Antibodies					
**Protein**	**Conjugate**	**Source Species**	**Dilution**	**Application**	**Supplier_Reference**
GCNA1/TRA98	-	rat	1:500	IF	Abcam_ab82527
WT1	-	rabbit	1:50	IF	Abcam_ab89901
TRIM71	-	rabbit	1:1000	WB	Sigma_HPA038142
DDX4/VASA	-	goat	1:500	WB	Santa Cruz_sc-48707
TUBULIN	-	mouse	1:2000	WB	Sigma_T9026
VINCULIN	-	mouse	1:2000	WB	Sigma_V9131
GAPDH	-	mouse	1:2000	WB	Acris_ACP001P
THY1.2/CD90.2	FITC	rat	1:400	FACS/MACS	Biolegend_140303
SSEA-1/CD15	Alexa 647	mouse	1:200	FACS	Biolegend_125607
ITGB3/CD61	PE-Cy7	armenian hamster	1:400	FACS	Biolegend_104317
**Supplementary Table 4B. Secondary Antibodies**					
**Protein**	**Conjugate**	**Source Species**	**Dilution**	**Application**	**Supplier_Reference**
rat IgG	Alexa 488	donkey	1:250	IF	Jackson ImmunoResearch_712-545-153
rabbit IgG	Cy3	donkey	1:400	IF	Jackson ImmunoResearch_711-165-152
rabbit IgG	HRP	goat	1:5000	WB	Cell Signaling Technologies_7074
mouse IgG	HRP	horse	1:5000	WB	Cell Signaling Technologies_7076
goat IgG	HRP	rabbit	1:10000	WB	Santa Cruz Biotechnology_sc-2768

Supplementary Table 4 (relative to Methods). Antibodies used for immunofluorescence (IF), western blot (WB), flow cytometry (FACS) and magnetic-assisted cell sorting (MACS).
